# VANGL2 inhibits antiviral IFN-I signaling by targeting TBK1 for autophagic degradation

**DOI:** 10.1126/sciadv.adg2339

**Published:** 2023-06-23

**Authors:** Zhiqiang Hu, Yingchao Xie, Jiansen Lu, Jianwu Yang, Jiahuan Zhang, Huaji Jiang, Hongyu Li, Yufeng Zhang, Dan Wu, Ke Zeng, Xiaochun Bai, Xiao Yu

**Affiliations:** ^1^ Department of Immunology, School of Basic Medical Sciences, Southern Medical University, Guangzhou, Guangdong, China.; ^2^ Department of Joint Surgery, the Fifth Affiliated Hospital of Southern Medical University, Guangzhou, Guangdong, China.; ^3^ Department of Cell Biology, School of Basic Medical Science, Southern Medical University, Guangzhou, Guangdong, China.; ^4^ Laboratory Medicine, Guangdong Provincial People’s Hospital (Guangdong Academy of Medical Sciences), Southern Medical University, Guangzhou, Guangdong, China.; ^5^ Yue Bei People’s Hospital Postdoctoral Innovation Practice Base, Southern Medical University, Guangzhou, Guangdong, China.; ^6^ Guangdong Provincial Key Lab of Single Cell Technology and Application, Southern Medical University, Guangzhou, Guangdong, China.

## Abstract

Stringent control of type I interferon (IFN-I) signaling is critical to potent innate immune responses against viral infection, yet the underlying molecular mechanisms are still elusive. Here, we found that Van Gogh–like 2 (VANGL2) acts as an IFN-inducible negative feedback regulator to suppress IFN-I signaling during vesicular stomatitis virus (VSV) infection. Mechanistically, VANGL2 interacted with TBK1 and promoted the selective autophagic degradation of TBK1 via K48-linked polyubiquitination at Lys^372^ by the E3 ligase TRIP, which serves as a recognition signal for the cargo receptor OPTN. Furthermore, myeloid-specific deletion of VANGL2 in mice showed enhanced IFN-I production against VSV infection and improved survival. In general, these findings revealed a negative feedback loop of IFN-I signaling through the VANGL2-TRIP-TBK1-OPTN axis and highlighted the cross-talk between IFN-I and autophagy in preventing viral infection. VANGL2 could be a potential clinical therapeutic target for viral infectious diseases, including COVID-19.

## Teaser

Classic PCP signaling factor VANGL2 links IFN-I and autophagy crosstalk in antiviral immunity.

## INTRODUCTION

Innate immunity is the critical first line of host defense against various pathogens, which requires complicated molecular interaction and extensive cellular responses. Discrimination of “non-self” pathogens from “self” components is the fundamental function of the innate immune system (*1*). During viral infection, pattern recognition receptors (PRRs), including Toll-like receptors, retinoic acid–inducible gene I (RIG-I)–like receptors (RLRs), Nod-like receptors (NLRs), and cyclic guanosine monophosphate–adenosine monophosphate synthase (cGAS), surveil the presence of viral nucleic acids and thereby trigger innate immune responses (*2*), such as type I interferon (IFN-I), nuclear factor κB (NF-κB), and inflammasome signaling pathways. After activation of Janus kinase/signal transducer and activator of transcription (JAK/STAT) signaling by IFN-I, IFN-stimulated genes (ISGs) generated lead to a great antiviral state, which is effective against virus infection (*3*). IFN-I signaling should be tightly controlled to balance eliminating invading pathogens and avoiding immune disorders. However, the mechanisms underlying the stringent control of IFN-I signaling are still not completely understood and need further dissection.

As the pivotal kinase in the IFN-I signaling pathway, TBK1 binds with stimulator of interferon genes (STING) or mitochondrial antiviral signaling protein (MAVS) to induce phosphorylation and activation of STING/MAVS and TBK1. Further recruitment and phosphorylation of IFN regulatory factor 3 (IRF3) and TBK1 lead to the induction of IFN-I (*4*). Different posttranslational modifications, including phosphorylation, ubiquitination, SUMOylation, and acetylation, have been reported to participate in the regulation of TBK1. The autophosphorylation of TBK1 at Ser^172^ is essential for its activation, and maintaining the stability of TBK1 is also critical for its function in IFN-I induction (*5*). Dynamic balance of ubiquitination and deubiquitination contributes to TBK1 stabilization. TRAF3-interacting protein 3 (TRAF3IP3), ubiquitin-specific protease 19 (USP19), ubiquitin-specific protease 38 (USP38), NLR family pyrin domain containing 4 (NLRP4), DELTEX family ubiquitin ligase 4 (DTX4), neural precursor cell expressed developmentally downregulated protein 4 (NEDD4), TRAF-interacting protein (TRIP), and other negative regulators have been found leading to K48/K63-linked polyubiquitination of TBK1 and its degradation (*6*–*9*). On the other hand, USP15 and Papain-like protease domain 2 (PLP2) deubiquitinate TBK1 and maintain its stability (*10*, *11*). However, multiple candidates might still influence these processes in physical and pathological conditions.

Autophagy is a catabolic degradative process delivering cytoplasmic components to the lysosome, which could be performed with nonselective bulk degradation or in a selective manner. Autophagy involves in various physiological processes, including starvation, cell differentiation and development, and degradation of aberrant structures, which ultimately maintain cellular homeostasis. Cell stress could activate autophagy during viral infection, which helps the innate immune system defend against invading viruses (*12*). TBK1 can regulate selective autophagy, especially for mitophagy and xenophagy, via phosphorylating autophagy modifiers and cargo receptors, including optineurin (OPTN), Nuclear Dot 52 kDa Protein (NDP52), Tax1 Binding Protein 1 (TAX1BP1), and p62/sequestosome 1 (p62) during autophagosome formation (*13*). A recent study found that TBK1 can be degraded through selective autophagy by NEDD4 (*7*), enlightening us about whether other molecules have similar regulation on TBK1.

Van Gogh–like 2 (VANGL2), a vertebrate counterpart of the *Drosophila melanogaster* Vang Gogh (Vang)/Strabismus (Stbm) protein, was discovered nearly two decades ago due to its core role in planar cell polarity (PCP) (*14*). Physically, VANGL2 is a membrane-embedded protein presented with four internal and contiguous transmembrane domains in the N-terminal half (*15*). Many studies have illustrated the central role of VANGL2 for PCP in signaling pathways such as the canonical Wnt and Rho pathways. In various mammals, VANGL2 regulates planar polarity, development, homeostasis, and repair of many organs like the brain, lungs, and kidneys (*16*, *17*). Besides its positive role, VANGL2 limits chaperone-mediated autophagy to balance osteogenic differentiation in mesenchymal stem cells (*18*). Another study found that VANGL2 is highly expressed in basal breast cancers, associated with poor prognosis, and implicated in tumor growth (*19*). These findings remind us of the harmful role of VANGL2 in related diseases. Nonetheless, the function of VANGL2 in immunity, especially antiviral innate immunity, has yet to be explored.

Our study found that VANGL2 suppressed the IFN-I signaling pathway and antiviral immune responses. During VSV infection, VANGL2 interacts with TBK1 and promotes K48-linked polyubiquitination at Lys^372^ of TBK1 via E3 ligase TRIP, which serves as a recognition signaling for cargo receptor OPTN and leads to the selective autophagic degradation of TBK1. Our work defines VANGL2 as an “immunological rheostat” in antivirus innate immunity and provides a potential therapeutic target in IFN-I–associated diseases.

## RESULTS

### VANGL2 inhibits the IFN-I immune response upon viral infection

**Fig. 1.**
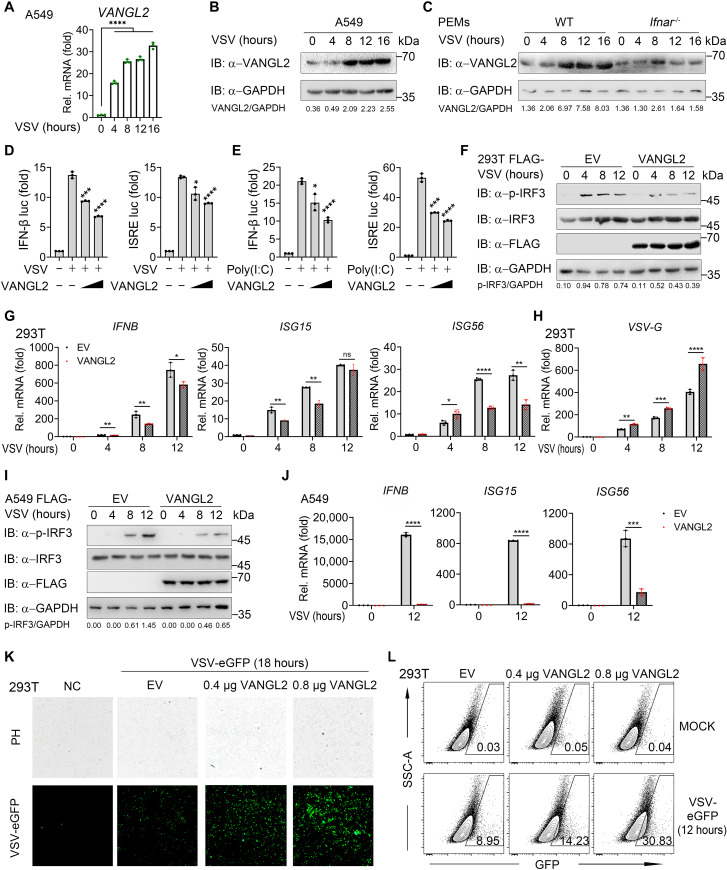
VANGL2 inhibits the IFN-I immune response upon viral infection. (**A** and **B**) Reverse transcription quantitative polymerase chain reaction (RT-qPCR) (A) and immunoblotting (B) analysis of VANGL2 mRNA and protein level change in A549 cells infected with vesicular stomatitis virus (VSV) [multiplicity of infection (MOI) of 0.5] for 0 to 16 hours. (**C**) Immunoblotting analysis of VANGL2 protein level changes in wild-type (WT) and *Ifnar^−/−^* peritoneal macrophages (PEMs) infected with VSV (MOI of 0.5) for the indicated times. (**D** and **E**) Luciferase reporter assays analyzing IFN-β or IFN-stimulated response element (ISRE) promoter activity of human embryonic kidney (HEK) 293T cells transfected with increasing amounts (wedge represents 300 and 500 ng) of HA-VANGL2 or empty vector (EV) for 24 hours, followed by treatment with or without VSV (MOI of 0.5) (D) or poly(I:C) (E) for 12 hours, respectively. (**F** to **J**) Immunoblotting analysis (F and I) of total and phosphorylated IRF3 and RT-PCR analysis (G), (H), and (J) of indicated gene expression in HEK293T (F) to (H) or A549 (I) and (J) cells transfected with FLAG-VANGL2 or EV for 24 hours, followed by VSV (MOI of 0.5) infection at indicated time points. GAPDH, glyceraldehyde-3-phosphate dehydrogenase. (**K** and **L**) Fluorescence microscopy analysis (K) and flow cytometric analysis (L) of the replication of VSV–enhanced green fluorescent protein (eGFP) in HEK293T cells transfected with EV or increasing HA-VANGL2 at indicated dose for 24 hours, followed by treatment with or without VSV-eGFP (MOI of 0.5) infection at indicated time points. Numbers adjacent to the outlined areas indicate percentages of GFP^+^ cells. NC, negative control. Data with error bars are represented as means ± SD. Each panel is a representative experiment of at least three independent biological replicates. *P < 0.05, **P < 0.01, ***P < 0.001, and ****P < 0.0001 as determined by unpaired Student’s *t* test. ns, not significant.

To explore the function of VANGL2 in immunity, we first compared the expression of VANGL2 in immune and nonimmune cells, including keratinocytes, mouse embryonic fibroblasts, HT29, A549, and human umbilical cord endothelial cells, which are known for the PCP function of VANGL2 (*20*). We found that VANGL2 is universally detected in immune cells (fig. S1, A to C), and vesicular stomatitis virus (VSV) infection led to an increase of VANGL2 in both A549 and THP-1 cells (Fig. 1, A and B, and fig. S1, D and E). Notably, administration of recombinant mouse IFN-β also induced *Vangl2* expression in mouse primary peritoneal macrophages (PEMs) (fig. S1F), and VSV infection–induced expression of VANGL2 was significantly reduced in PEMs from *Ifnar*-deficient (*Ifnar*^−/−^) mice (Fig. 1C and fig. S1G). These results suggest that VANGL2 expression is induced by virus infection in an IFN-I–dependent way.

Given that, we next sought to evaluate the function of VANGL2 in antiviral immune responses. By overexpressing VANGL2 in 293T cells, we found that VANGL2 markedly suppressed activation of IFN-β– and IFN-stimulated response element (ISRE)–responsive reporters induced by VSV, intracellular polyinosinic-polycytidylic acid [poly(I:C)], exogenous genomic DNA (gDNA), and RNA (Fig. 1, D and E, and fig. S1, H and I). Consistently, ectopic expression of VANGL2 greatly inhibited IRF3 phosphorylation and transcription of antiviral genes, including *IFNB*, *ISG15*, and *ISG56*, after VSV infection in 293T and A549 cells (Fig. 1, F, G, I, and J), which, nevertheless, promoted viral replication (Fig. 1H). Moreover, we observed that the viral burden was significantly promoted in 293T cells transfected with an increasing amount of exogenous *VANGL2* plasmids, which were consistent with the reduced expression of IFNs (Fig. 1, K and L, and fig. S1, J and K). Additionally, a previous publication reported that TRAF3IP3 could also negatively regulate anti-VSV IFN-I immunity. To probe whether there are relative contributions of TRAF3IP3 and VANGL2 to the inhibition of antiviral IFN-I signaling, we overexpressed or silenced VANGL2, TRAF3IP3, or both in the VSV-infected 293T and A549 cells. We found that VANGL2 and TRAF3IP3 have a synergistic effect in suppressing IFN-I signaling (fig. S1, L to O). In general, these results suggest that IFN-induced VANGL2 during virus infection effectively inhibits IFN-I signaling and antiviral immunity.

### VANGL2 deficiency strengthens the IFN-I response and antiviral immunity

**Fig. 2.**
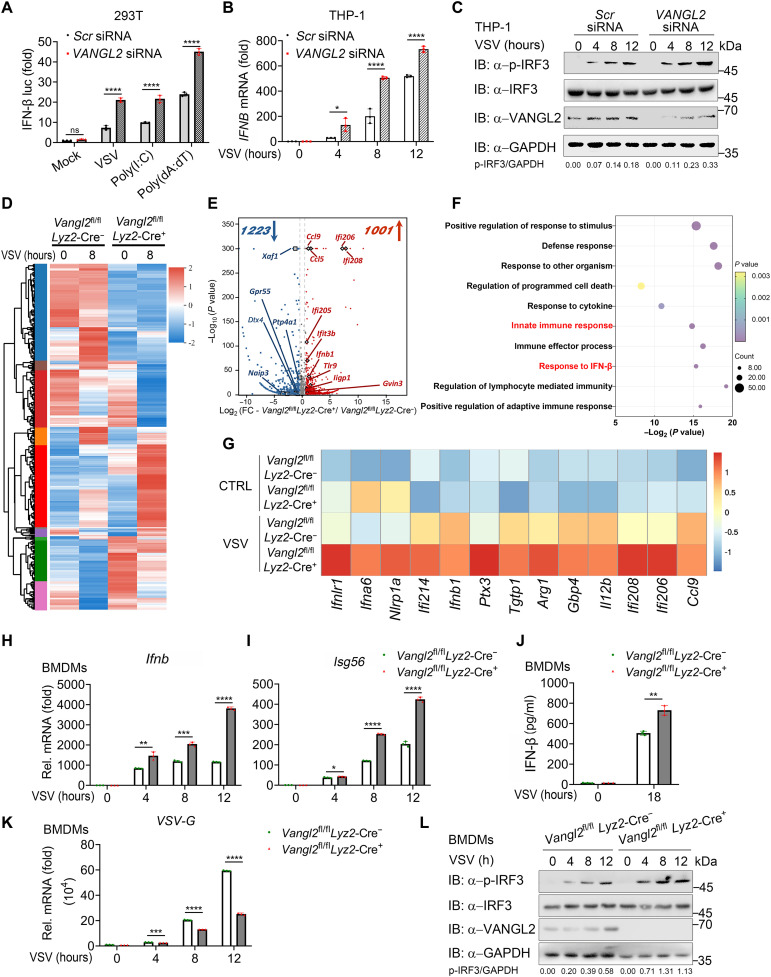
VANGL2 deficiency strengthens IFN-I response and antiviral immunity. (**A**) Luciferase activity in HEK293T cells transfected with scrambled (*Scr*) small interfering RNA (siRNA) or siRNA-targeting *VANGL2* for 24 hours and then transfected with an IFN-β luciferase (IFN-β luc) for 24 hours, followed by treatment with or without VSV (MOI of 0.5), poly(I:C), or poly (dA:dT) for 12 hours. (**B** and **C**) RT-PCR (B) and immunoblotting (C) analysis of VSV (MOI of 0.5)–infected THP-1 cells transfected with *Scr* siRNA or *VANGL2*-specific siRNA at indicated time points. (**D**) Heatmap view of top and bottom gene list of RNA-sequence data sets. Microarray analysis for total RNA was performed for *Vangl2*^fl/fl^ *Lyz2*-Cre^−^ and *Vangl2*^fl/fl^ *Lyz2*-Cre^+^ bone marrow–derived macrophages (BMDMs) with or without VSV infection. (**E**) VANGL2 regulates antiviral response-relevant target genes, presented as a volcano plot of genes with differential expression after VSV infection in *Vangl2*^fl/fl^ *Lyz2*-Cre^−^ and *Vangl2*^fl/fl^ *Lyz2*-Cre^+^ BMDMs. FC, fold change. (**F**) Gene ontology (GO) enrichment analysis of the VANGL2-dependent genes in (E) (−log_2_ *P* values). (**G**) Heatmap showing the change of indicated ISGs in *Vangl2*^fl/fl^ *Lyz2*-Cre^−^ and *Vangl2*^fl/fl^ *Lyz2*-Cre^+^ BMDMs with or without VSV infection. (**H** to **L**) RT-PCR analysis of *Ifnb* (H), *Isg56* (I), and *VSV-G* (K) mRNA expression, IFN-β enzyme-linked immunosorbent assay (ELISA) (J), and immunoblotting (L) analysis of total and phosphorylated IRF3 using *Vangl2*^fl/fl^ *Lyz2*-Cre^−^ and *Vangl2*^fl/fl^ *Lyz2*-Cre^+^ BMDMs infected with VSV (MOI of 0.5) for the indicated times. Data with error bars are represented as means ± SD. Each panel is a representative experiment of at least three independent biological replicates. *P < 0.05, **P < 0.01, ***P < 0.001, and ****P < 0.0001 as determined by unpaired Student’s *t* test.

Next, to further determine the function of VANGL2 in regulating the IFN-I signaling pathway, we silenced the expression of VANGL2 by small interfering RNAs (siRNAs) in 293T and performed the IFN-β luciferase reporter assay in 293T cells after stimulation of VSV, poly(I:C), or polyinosinic-polycytidylic acid [poly(dA:dT)] and found that knockdown of VANGL2 acted to significantly increase the activity of IFN-β promoter (Fig. 2A). We further knocked down VANGL2 and found that silencing of VANGL2 could promote transcription of *IFNB*, along with the phosphorylation of IRF3 in both THP-1 cells and human peripheral blood mononuclear cells (PBMCs) (Fig. 2, B and C, and fig. S2A).

To further elucidate the function of VANGL2 in vivo, we generated *Vangl2*^fl/fl^*Lyz2*-Cre^+^ mice, with VANGL2 expression specifically abolished in myeloid cells (fig. S2, B to D). We then performed the transcriptomic analysis to identify the signaling pathways that VANGL2 mainly regulates in VSV-treated bone marrow–derived macrophages (BMDMs) from control and *Vangl2*–conditional knockout (CKO) mice. Compared with the control group, infection of BMDMs from *Vangl2*-CKO mice resulted in 2224 differentially expressed genes (1001 up-regulated and 1223 down-regulated) (Fig. 2, D and E). Gene ontology (GO) analysis further revealed that deletion of VANGL2 resulted in the up-regulation of expression of numerous genes involved in multiple immune signaling pathways, including “response to IFN-β” and “innate immune response” (Fig. 2F). In addition, we also found expression of several representative genes associated with antiviral immune response elevated in BMDMs from *Vangl2*-CKO mice after VSV infection (Fig. 2G). Notably, our results of the reverse transcription quantitative polymerase chain reaction (RT-qPCR) and Western blot analysis confirmed that IFN-I signaling and antiviral response were significantly enhanced in the VANGL2-deficient BMDMs (Fig. 2, H to L), which were also verified in PEMs (fig. S2, E to H) and peritoneal neutrophils (NEs) (fig. S2, I to K). These results suggest that VANGL2 deficiency potentiates antiviral IFN-I response and antiviral immunity.

### VANGL2 targets TBK1

**Fig. 3.**
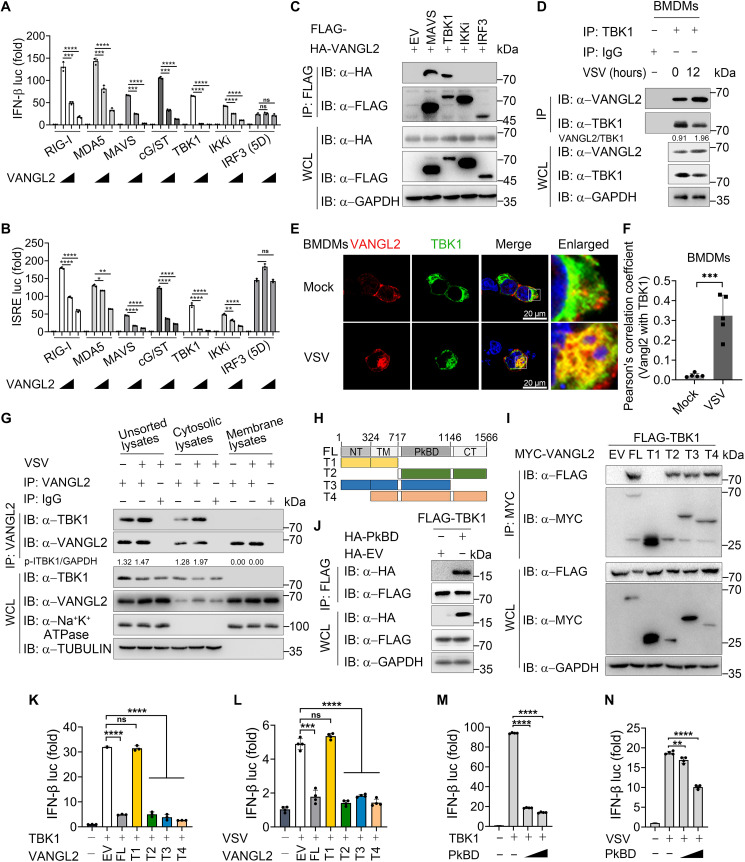
VANGL2 targets at TBK1. (**A** and **B**) Luciferase reporter assays analyzing IFN-β (A) or ISRE (B) promoter activity of HEK293T cells transfected with the Flag-tagged indicated plasmids along with EV or increasing amounts (from 100 to 200 ng) of HA-VANGL2. (**C**) Co-immunoprecipitation (co-IP; with anti-FLAG) and immunoblotting analysis using protein lysates of HEK293T cells transfected with indicated plasmids. WCL, whole cell lysates. (**D**) Co-IP (with anti-TBK1) and immunoblotting analysis using endogenous proteins lysates of control and VSV (MOI of 0.5, 12 hours)–infected BMDMs. (**E**) Control and VSV (MOI of 0.5, 12 hours)–infected BMDMs were labeled with the indicated antibodies and analyzed via confocal microscopy. Red, VANGL2 signal; green, TBK1 signal; blue, 4′,6-diamidino-2-phenylindole (DAPI). Scale bars, 20 μm. (**F**) Quantitative analysis of the colocalization in (E). (**G**) Co-IP (with anti-VANGL2) and immunoblotting analysis using unsorted, cytosolic, and membrane lysates of THP-1 cells with or without VSV infection for 12 hours. (**H**) Schematic mapping of VANGL2. (**I**) Co-IP and immunoblotting analysis using lysates from HEK293T cells transfected with MYC-VANGL2 and its truncations along with FLAG-TBK1. (**J**) Co-IP (with anti-FLAG) and immunoblotting analysis using lysates from HEK293T cells transfected with vectors for HA-PKBD along with FLAG-TBK1. PkBD, the Prickle-binding domain. (**K** and **L**) Luciferase reporter assays analyzing IFN-β promoter activity of HEK293T cells transfected with MYC-VANGL2 and its deletions along with FLAG-TBK1 (K) or infected with VSV (L) (MOI of 0.5). (**M** and **N**) Luciferase reporter assays analyzing IFN-β promoter activity of HEK293T cells transfected with increasing amounts (from 300 to 500 ng) of HA-PKBD or EV along with FLAG-TBK1 (M) or infected with VSV (N) (MOI of 0.5). Data with error bars are represented as means ± SD. Each panel is a representative experiment of at least three independent biological replicates. *P < 0.05, **P < 0.01, ***P < 0.001, and ****P < 0.0001 as determined by unpaired Student’s *t* test.

The cGAS-STING and RLR-MAVS signaling axis were identified for their essential role in antiviral defense and IFN-I induction (*21*, *22*). To further investigate molecular mechanisms behind the regulation of VANGL2 on the IFN-I signaling pathway, we performed a screen using dual luciferase reporter assay and found that an increasing VANGL2 overexpression significantly reduced IFN-β and ISRE promotor activity triggered by cotransfecting plasmids expressing RIG-I, MDA5, MAVS, cGAS plus STING, TBK1, or inducible IκB kinase (IKKi), except for IRF3 (5D) (Fig. 3, A and B). We then performed a co-immunoprecipitation (co-IP) assay with the overexpressing VANGL2 and these members in IFN-I signaling. Immunoblotting results showed that VANGL2 interacted with MAVS and TBK1 obviously (Fig. 3C). TBK1 is the downstream of MAVS and the negative regulation of VANGL2 to IFN-I at MAVS relies on TBK1, so we mainly focused on the effect that VANGL2 targets on TBK1. Endogenous co-IP assay showed the interaction between VANGL2 and TBK1 enhanced after VSV infection in BMDMs and A549 cells (Fig. 3D and fig. S3, A to C). Consistently, confocal microscopy analysis showed that VANGL2 had more colocalization with TBK1 in BMDMs and PEMs after VSV infection (Fig. 3, E and F, and fig. S3, D and E). VANGL2, a membrane-embedded protein, is mostly found in the cell membrane, cytoplasm, endoplasmic reticulum, and nucleus. (*16*). To further explore where the VANGL2-TBK1 complex is subcellular located, we separated cytosolic fractions and cytomembrane fractions as previously reported (*23*, *24*). Then, both lysates were generated and subjected to co-IP assay. Consistent with previous studies that TBK1 is a cytoplasmic protein, we only detected the TBK1-VANGL2 complex existing in the cytoplasm, and the interaction was enhanced during VSV infection (Fig. 3G).

Furthermore, VANGL2 domain mapping analysis showed that VANGL2 is composed of an N-terminal domain (NT; 1 to 324), transmembrane domain (TM; 325 to 717), and C-terminal domain (CT; 718 to 1556), which contains a Prickle/cadherin-binding domain (PkBD; 717 to 1146) (Fig. 3H). We generated four truncated mutants of VANGL2 to inquire which domain(s) is vital for interaction with TBK1. Co-IP experiments showed that VANGL2 interacted with TBK1 through its PkBD (Fig. 3, I and J). Moreover, overexpression of these truncates of VANGL2 suggested that the PkBD suppresses the activation of the IFN-β luciferase reporter triggered by TBK1, VSV, exogenous RNAs, or poly(I:C) (Fig. 3, K to N, and fig. S3, F and G). These results reveal that VANGL2 targets at the TBK1 level through its PkBD, which is critical for suppressing IFN-I signaling. To further investigate the interaction between VANGL2 and TBK1, we generated full-length or truncated TBK1 plasmids, and complementary binding studies using truncated TBK1 constructs showed that VANGL2 only interacted with the ubiquitin-like domain (ULD) domain of TBK1 (fig. S3H). These results suggest that VANGL2 interacts with TBK1 to suppress IFN-I signaling.

### VANGL2 promotes the autophagic degradation of TBK1

**Fig. 4.**
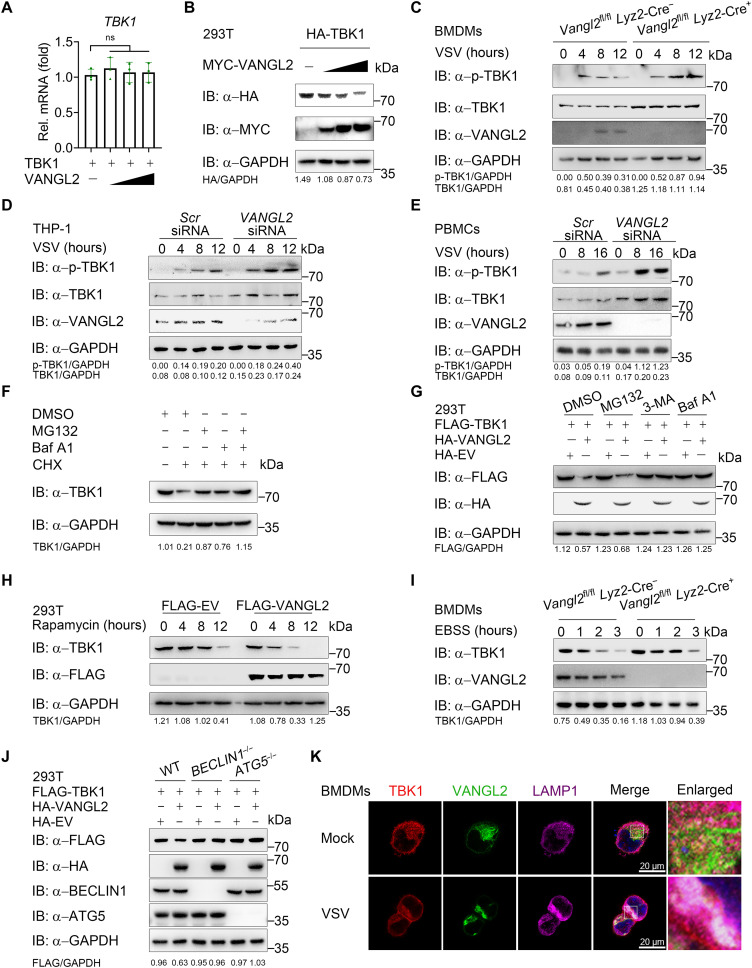
VANGL2 promotes the autophagic degradation of TBK1. (**A** and **B**) RT-qPCR (A) and immunoblotting (B) analysis of TBK1 mRNA and protein level change in HEK293T cells transfected with HA-TBK1, and increasing amounts of MYC-VANGL2. (**C**) Immunoblotting analysis of total and phosphorylated TBK1 using *Vangl2*^fl/fl^ *Lyz2*-Cre^−^ and *Vangl2*^fl/fl^ *Lyz2*-Cre^+^ BMDMs infected with VSV (MOI of 0.5) for the indicated times. (**D** and **E**) Immunoblotting analysis of total and phosphorylated TBK1 using THP-1 (D) or PBMCs (E) transfected with *Scr* siRNA or *VANGL2* siRNA for 24 hours and then infected with VSV (MOI of 0.5) for indicated time. (**F**) Immunoblotting analysis of protein extracts of HEK293T cells treated with CHX (100 μg/ml) for 12 hours, followed by treatment with MG132 (10 μM), Baf A1 (0.2 μM), or both for 6 hours. (**G**) Immunoblotting analysis of HEK293T cells transfected with indicated plasmids for 24 hours, followed by treatment with MG132 (10 μM), 3-MA (10 mM), or Baf A1 (0.2 μM) for 6 hours. (**H**) Immunoblotting analysis using lysates from HEK293T cells transfected with FLAG-VANGL2 or FLAG-EV for 24 hours, followed by treatment with rapamycin (250 nM) for indicated time. (**I**) Immunoblotting analysis using lysates from *Vangl2*^fl/fl^ *Lyz2*-Cre^−^ and *Vangl2*^fl/fl^ *Lyz2*-Cre^+^ BMDMs treated with EBSS for indicated time. (**J**) Immunoblotting analysis of WT, *BECLIN*-KO, or *ATG5*-KO HEK293T cells transfected with indicated plasmids. (**K**) Control and VSV (MOI of 0.5, 12 hours)–infected BMDMs were labeled with the indicated specific antibodies and analyzed via confocal microscopy. Red, VANGL2 signal; green, TBK1 signal; violet, LAMP1 signal; blue, DAPI. Scale bars, 20 μm. Data with error bars are represented as means ± SD. Each panel is a representative experiment of at least three independent biological replicates.

Next, to determine additional mechanisms by which VANGL2 regulates TBK1 through their interaction, we transfected TBK1 along with an increased amount of VANGL2 in 293T cells and found that VANGL2 markedly decreased the protein level, but not the mRNA level, of TBK1 in a dose-dependent manner (Fig. 4, A and B). In contrast to the impaired TBK1 protein stability, but not other molecules in IFN-I signaling (fig. S4A), this suggests that VANGL2 especially down-regulates TBK1 via posttranscriptional modification (PTM). We next infected BMDMs and PEMs from control and VANGL2-CKO mice with VSV and sought to determine the role of VANGL2 in mediating the degradation of endogenous TBK1 during viral infection and found that VANGL2 deficiency could recover the impaired stability of TBK1 and IFN-I signaling (Fig. 4C and fig. S4B). Similar results were confirmed in THP-1 cells and human PBMCs when silencing VANGL2 expression by siRNA (Fig. 4, D and E). Moreover, KO of VANGL2 also recovered the reduction of TBK1 in BMDMs treated with cycloheximide (CHX), with or without VSV infection (fig. S4C). Consistently, VANGL2-overexpressing A549 cells infected with VSV hold an enhanced degradation of endogenous TBK1 compared to the empty vector–transfected control cells (fig. S4D), suggesting VANGL2-induced degradation of TBK1 to inhibit IFN-I signaling.

Most literature showed that the degradation of TBK1 is relied on the proteasome, except for a few recent studies that indicated that USP19, NEDD4, and severe acute respiratory syndrome coronavirus 2 (SARS-CoV-2) nonstructural protein 13 (NSP13) could induce the autophagic degradation of TBK1 (*7*, *25*, *26*). To further confirm that both protein degradation systems contribute to TBK1 degradation, we treated 293T cells with CHX and then used MG132, which effectively blocks the proteolytic activity of the 26S proteasome complex, 3-methyladenine (3-MA) that is a widely used inhibitor of autophagy via its inhibitory effect on class III phosphatidylinositol 3-kinase, or both to rescue the degraded TBK1. Compared to the classical proteasome-induced inhibitor of nuclear factor κBα degradation (*27*) and autophagy-induced NDP52 degradation (fig. S4, E and F) (*28*), we found that the damaged protein level of TBK1 could be partially rescued by MG132 or 3-MA treatment and that the combination of MG132 and 3-MA could fully rescue the degraded TBK1 (Fig. 4F), which indicated that TBK1 holds both proteasomal and autophagic degradation.

VANGL2 has been reported to induce proteasomal degradation (*29*). We attempted to verify whether this regulative way is also applied to TBK1. After treatment of 293T cells overexpressing VANGL2 and TBK1 with MG132, 3-MA, or bafilomycin A1 (Baf A1) that blocks autophagosome-lysosome fusion and inhibits acidification and protein degradation in lysosomes, or Caspase inhibitors, Z-VAD-FMK (Z-VAD) and VX-765, we found that the degradation of TBK1 induced by VANGL2 could be rescued by the lysosome inhibitor 3-MA and Baf A1, but not by proteasome inhibitor MG132 or caspase inhibitors (Fig. 4G and fig. S4G). Moreover, overexpression of VANGL2 accelerated the autophagic degradation of TBK1 induced by rapamycin, while VANGL2 KO rescued the TBK1 degradation in Earle's Balanced Salt Solution (EBSS)–treated BMDMs (Fig. 4, H and I).

The above results showed that autophagic inhibitors could rescue VANGL2-induced TBK1 degradation, and we further attempt to verify whether this phenomenon could also exist in autophagic gene-deficient cells. Consistently, we found that TBK1 was not degraded in *BECLIN1-* and *ATG5*-KO cells in CHX-treated physiological conditions (fig. S4, H and I). Furthermore, we transfected wild-type (WT), *BECN1*-KO, and *ATG5*-KO 293T cells with VANGL2 and found that the degradation of TBK1 triggered by VANGL2 was abrogated in *BECLIN1-* and *ATG5*-KO cells (Fig. 4J). Fusion of autophagosomes and lysosomes is necessary for the degradation of autophagic substrates (*30*), and VANGL2 was reported to be related to the lysosome (*18*). Next, we performed confocal analysis and found that puncta of VANGL2/TBK1 have a strong colocalization with the lysosome-associated membrane protein 1 (LAMP1) after VSV infection, which further explained the above findings (Fig. 4K and fig. S4J). Negative regulator like NEDD4 specifically promotes the degradation of activated phospho-TBK1 (p-TBK1) after viral infection (*7*). Here, we cotransfected 293T cells with VANGL2 and WT TBK1 or its mutant (S172A) that abolishes autophosphorylation of TBK1 (*5*), and we found that VANGL2 could still bind to and degrade TBK1 S172 mutant, suggesting that the function of VANGL2 on TBK1 is not dependent on whether TBK1 is activated (fig. S4, K and L). These results suggest that VANGL2 promotes the autophagic degradation of TBK1 during VSV infection.

### VANGL2 enhances the recognition of TBK1 by cargo receptor OPTN

**Fig. 5.**
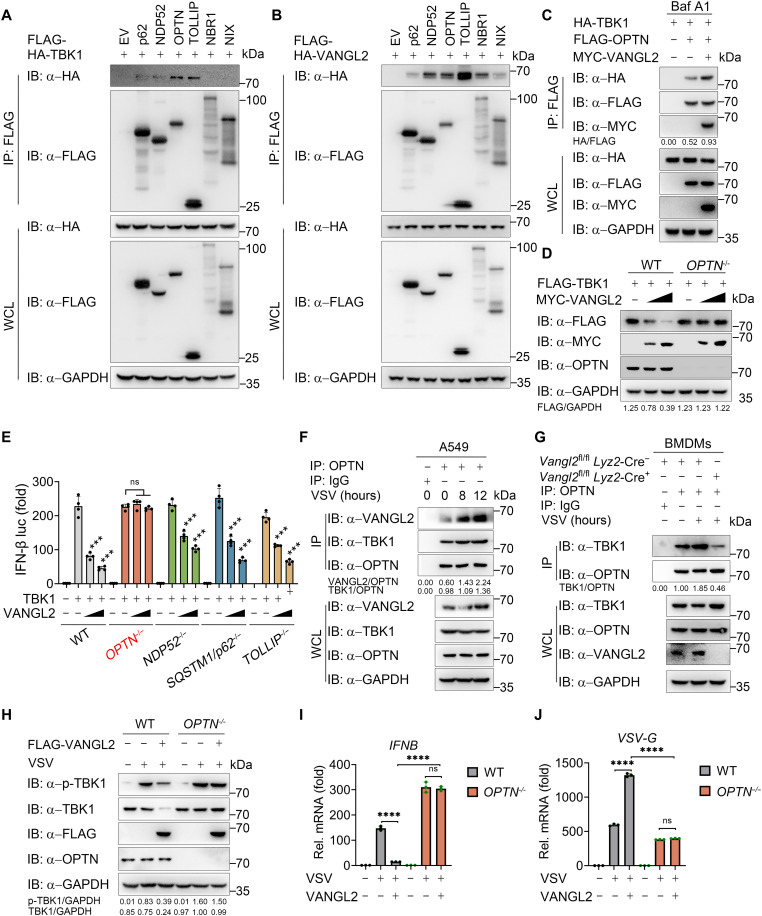
VANGL2 enhances the recognition of TBK1 by cargo receptor OPTN. (**A** and **B**) Co-IP (with anti-FLAG) and immunoblotting analysis using lysates from HEK293T cells transfected with indicated Flag-tagged cargo receptors along with HA-TBK1 (A) or HA-VANGL2 (B). (**C**) Cell lysates were harvested after Baf A1 (0.2 μM) treatment (6 hours) for co-IP (with anti-FLAG) and immunoblotting analysis of HEK293T cells transfected with HA-TBK1, FLAG-OPTN, and MYC-VANGL2. (**D**) Immunoblotting analysis of WT and *OPTN^−/−^* HEK293T cells transfected with FLAG-TBK1 and MYC-EV or MYC-VANGL2 for 24 hours. (**E**) Luciferase reporter assays analyzing IFN-β promoter activity of WT, *OPTN*-KO, *NDP52-*KO, *p62*-KO, or *TOLLIP*-KO HEK293T cells transfected with FLAG-TBK1, together with increasing amounts (wedge represents 300 and 500 ng) of HA-VANGL2 for 24 hours. (**F** and **G**) A549 (F) or BMDMs (G) cells were infected with VSV (MOI of 0.5), and protein lysates were harvested for IP using an anti-OPTN antibody. (**H** to **J**) Immunoblotting (H) and RT-PCR (I) and (J) analysis of WT and *OPTN*-KO HEK293T cells transfected with FLAG-EV or FLAG-VANGL2 for 24 hours, followed by treatment with or without VSV (MOI of 0.5) infection for 12 hours. Data with error bars are represented as means ± SD. Each panel is a representative experiment of at least three independent biological replicates. ***P < 0.001 and ****P < 0.0001 as determined by unpaired Student’s *t* test.

Substantial evidence reveals that the cargo receptor is the key element that targets protein complexes, aggregates, and whole organelles into lysosomes and then recognizes signals on cargoes to deliver them to autophagosomes (*31*). To identify the responsible cargo receptor contributing to the VANGL2-induced autophagic degradation of TBK1, we first performed co-IP assays using several receptors, including p62, NDP52, OPTN, toll interacting protein (TOLLIP), neighbor of BRCA1 (NBR1), and Nip-like protein X (NIX), cotransfected with TBK1. We found that TBK1 interacted with the p62, NDP52, OPTN, and TOLLIP (Fig. 5A). Meanwhile, we also found that VANGL2 has an interaction with all cargo receptors above (Fig. 5B). Further studies showed that the interaction between TBK1 and OPTN was strengthened when VANGL2 was overexpressed, whereas the association between TBK1 and NDP52, p62, or TOLLIP was not altered (Fig. 5C and fig. S5, A to C). Moreover, VANGL2 failed to decrease TBK1 protein level or impair IFN-I signaling activation in *OPTN*-KO 293T cells, but not in *NDP52-*, *p62-*, or *TOLLIP*-KO cells (Fig. 5, D and E, and fig. S5, D and E). In addition, compared to WT cells, TBK1 stability was no longer damaged by CHX or rapamycin in *OPTN*-KO 293T cells (fig. S5, F and G). Together, these results indicate that OPTN functions as the cargo receptor for the autophagic degradation of TBK1. To further investigate the role of VANGL2 in the association between OPTN and TBK1, we first found that VANGL2 could interact with OPTN, and this interaction was enhanced in A549 cells during VSV infection (Fig. 5F), which suggested that VANGL2, TBK1, and OPTN formed a complex. Further studies showed that VANGL2 deficiency observably weakened the association of endogenous TBK1 and OPTN in primary BMDMs and PEMs (Fig. 5G and fig. S5H). Furthermore, the functional assay showed that IFN-I response in VSV-infected *OPTN*-KO 293T cells was no longer impaired by exogenous VANGL2 overexpression (Fig. 5, H and I), which was also presented in slower virus replication (Fig. 5J). These results suggest that VANGL2 facilitates the interaction between TBK1 and OPTN for selective autophagic degradation.

### VANGL2 promotes the K48-linked polyubiquitination of TBK1 through TRIP

**Fig. 6.**
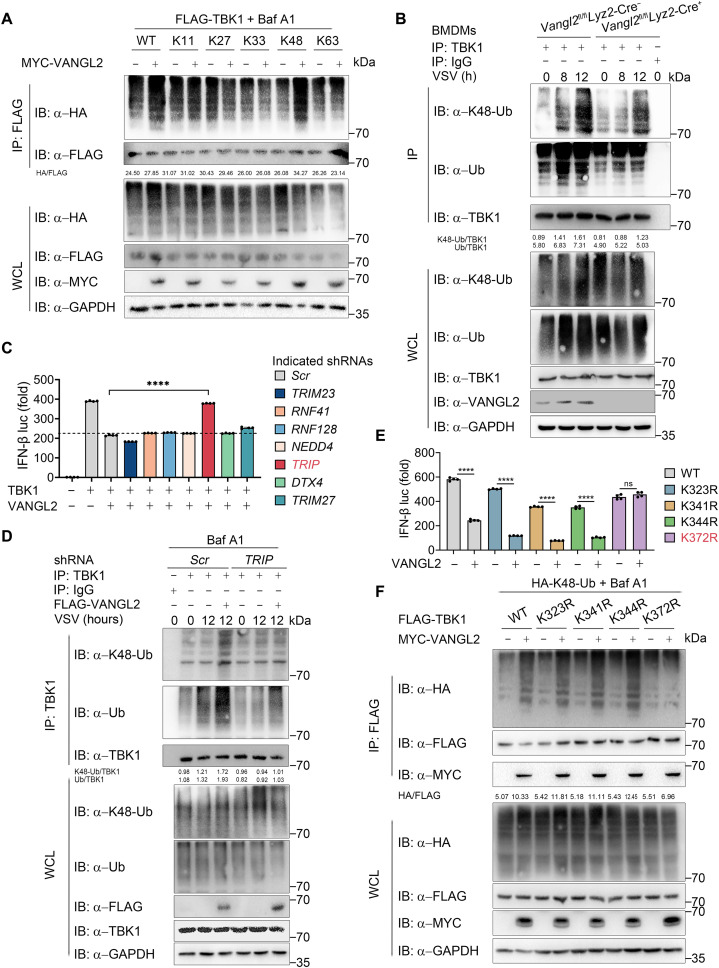
VANGL2 promotes the K48-linked polyubiquitination of TBK1 at Lys^372^ through TRIP. (**A**) HEK293T cells were transfected with FLAG-TBK1 and HA-tagged WT ubiquitin (HA-Ub) or its mutants, together with MYC-EV or MYC-VANGL2 for 24 hours, followed by treatment with Baf A1 (0.2 μM) for 6 hours, followed by co-immunoprecipitated with anti-Flag beads and immunoblotted with anti-HA antibody. (**B**) IP (with anti-TBK1) and immunoblotting analysis using indicated antibodies of *Vangl2*^fl/fl^ *Lyz2*-Cre^−^ and *Vangl2*^fl/fl^ *Lyz2*-Cre^+^ BMDMs infected with VSV (MOI of 0.5) for the indicated times. (**C**) Luciferase reporter assays analyzing IFN-β promoter activity of HEK293T cells transfected with *Scr* shRNA or other E3 ligase–specific shRNAs for 24 hours, followed by transfected with FLAG-TBK1, together with EV or HA-VANGL2 for 24 hours. (**D**) HEK293T cells were transfected with *Scr* shRNA or *TRIP-*specific shRNA for 24 hours, followed by transfected with FLAG-EV or FLAG-VANGL2 for 24 hours; protein lysates were harvested after VSV (MOI of 0.5) infection for 12 hours and Baf A1 (0.2 μM) treatment for 6 hours for IP (with anti-TBK1) and immunoblotting analysis using indicated antibodies. (**E**) Luciferase reporter assays analyzing IFN-β promoter activity of HEK293T cells transfected with WT FLAG-TBK1 or its K323R, K341R, K344R, and K372R mutant, together with MYC-EV or MYC-VANGL2 for 24 hours. (**F**) Co-IP (with anti-FLAG) and immunoblotting analysis of HEK293T cells transfected with WT FLAG-TBK1 or its K323R, K341R, K344R, and K372R mutant, together with HA-K48–linked ubiquitin and MYC-EV or MYC-VANGL2; cell lysates were harvested after Baf A1 (0.2 μM) treatment for 6 hours. Data with error bars are represented as means ± SD. Each panel is a representative experiment of at least three independent biological replicates. ****P < 0.0001 as determined by unpaired Student’s *t* test.

Ubiquitination of TBK1 is an important step in its autophagic degradation. To verify whether VANGL2 affects TBK1 ubiquitination, we co-overexpressed TBK1 in the presence of VANGL2 or not, which is also together with various ubiquitin linkages. The immunoblotting result showed that VANGL2 characteristically enhanced K48-linked (K48-only ubiquitin mutant) polyubiquitination of TBK1 but not others (Fig. 6A). K48-linked polyubiquitination has been identified vitally for strengthening its degradation (*9*, *32*). Consistently, we found that VANGL2 deficiency in BMDMs markedly decreased both total and K48-linked polyubiquitination of TBK1 induced by VSV infection (Fig. 6B). VANGL2 is not an E3 ubiquitin ligase, so we hypothesized that VANGL2 might function as a scaffold protein to link TBK1 and its E3 ligases for ubiquitination and degradation. E3 ligases including TRIM23, RNF41, RNF128, NEX4, TRIP, DTX4, and TRIM27 have been indicated participating in polyubiquitination and degradation of TBK1 (*6*–*9*, *33*, *34*). We then silenced the expression of these E3 ligases with short hairpin–mediated RNA (shRNA) (fig. S6A) and detected that silencing of TRIP rescued VANGL2-mediated inhibition on TBK1-induced IFN-β promoter activity (Fig. 6C). We also found that TRIP knockdown could block VANGL2-mediated TBK1 degradation, while overexpression of TRIP aggravated VANGL2-induced TBK1 degradation (fig. S6, B and C). Moreover, we confirmed that VANGL2 could interact with TRIP and promote the interaction between TRIP and TBK1 (fig. S6, D and E). To explore the mechanism behind this, we found that both total and K48-linked polyubiquitination of TBK1 were not altered by VANGL2 when the TRIP expression was silenced after VSV infection (Fig. 6D). Consistently, we then infected 293T cells with VSV and found that decreased TRIP expression potentiates antiviral IFN-I response (fig. S6, F and G). These results suggest that TRIP is essential for catalyzing the K48-linked polyubiquitination of TBK1, thus inhibiting antiviral IFN-I signaling.

Lysine (Lys) residues are key features of ubiquitin, which give rise to isopeptide-linked ubiquitin chains and are necessary for additional posttranslational modifications (*35*). We next attempted to identify the specific sites of TRIP-mediated ubiquitination in TBK1. Because ULD of TBK1 is vital for binding VANGL2 (fig. S3G) and the degradation and K48-linked polyubiquitination of TBK1 mutant containing the ULD domain, but not kinase domain (KD) or coiled coil (CC) domain, were altered by VANGL2 (fig. S6, H to J), this suggests that ULD domain of TBK1 is necessary for VANGL2-mediated degradation and ubiquitination of TBK1. The ULD domain of TBK1 contains four Lys residues: Lys^323^, Lys^341^, Lys^344^, and Lys^372^ (*7*). To explore which Lys residue is critical for VANGL2-mediated TBK1 ubiquitination, we mutated them from K to R. Further experiments showed that VANGL2 failed to promote the degradation of TBK1 K372R mutant (fig. S6K) and that VANGL2 could not restrict the activation of IFN-I signaling that TBK1 K372R mutant induced (Fig. 6E). Consistently, VANGL2 no longer facilitated the K48-linked polyubiquitination of TBK1 K372R mutant (Fig. 6F). Collectively, these results suggested that VANGL2-induced autophagic degradation of TBK1 is dependent on K48-linked polyubiquitination of TBK1 at Lys^372^.

### VANGL2-deficient mice exhibit stronger resistance to VSV infection

**Fig. 7.**
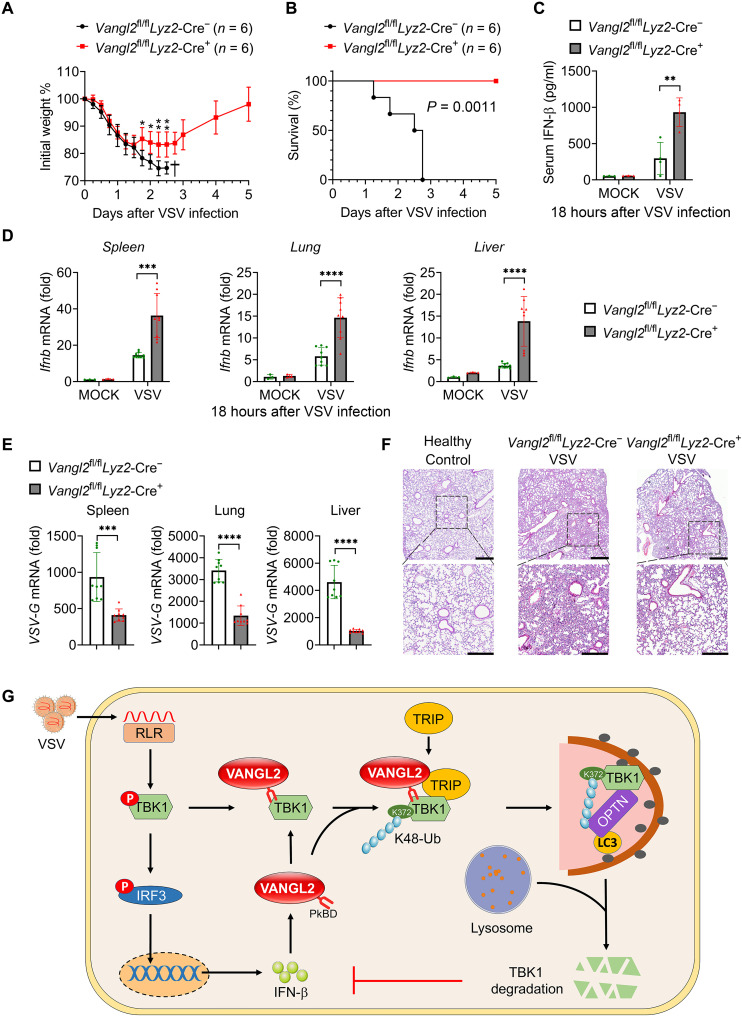
The effect of VANGL2 ablation on antiviral immunity in vivo. (**A** and **B**) Weight (A) and survival (B) of *Vangl2*^fl/fl^ *Lyz2*-Cre^−^ and *Vangl2*^fl/fl^ *Lyz2*-Cre^+^ mice (*n* = 6 mice per group) after intraperitoneal injection of VSV [1 × 10^8^ plaque-forming units (PFU) per mouse]. (**C**) ELISA for IFN-β in serum of *Vangl2*^fl/fl^ *Lyz2*-Cre^−^ and *Vangl2*^fl/fl^ *Lyz2*-Cre^+^ mice treated with phosphate-buffered saline (PBS) or infected with VSV (1 × 10^8^ PFU per mouse) via intraperitoneal injection for 18 hours. (**D** and **E**) RT-PCR analysis of *Ifnb* (D) or *VSV-G* (E) mRNA in the spleen (left), lungs (center), and liver (right) from mice, as in (C). (**F**) Representative hematoxylin and eosin–stained images of lung sections from mice as in (C). Scale bars, 50 μm. (**G**) Graphical abstract to illustrate how VANGL2 negatively regulates IFN-I signaling upon virus infection. Data with error bars are represented as means ± SD. Each panel is a representative experiment of at least three independent biological replicates. *P < 0.05, **P < 0.01, ***P < 0.001, and ****P < 0.0001 as determined by unpaired Student’s *t* test.

Last, to further determine the role of VANGL2 in host defense against viral infection in vivo, we infected the control and *Vangl2*-CKO mice using VSV by intraperitoneal injection. Notably, the *Vangl2*-CKO mice presented less susceptibility and slighter weight loss to VSV-induced lethality than the control group (Fig. 7, A and B). The transcriptional levels of *Ifnb* and *Isg56* were significantly higher in the spleen, lung, and liver and increased IFN-β production in serum from *Vangl2*-CKO mice compared to the control group at 18 hours after VSV infection (Fig. 7, C and D, and fig. S7A). Consistent with the higher amounts of IFNs, the viral proliferation was significantly weakened (Fig. 7E), and, consequently, the severity of pulmonary inflammation was significantly moderated in *Vangl2*-CKO mice (Fig. 7F). Collectively, these data demonstrate that VANGL2 is a negative regulator of IFN-I signaling in response to VSV infection (Fig. 7G).

## DISCUSSION

IFN-I is essential for host defense against invading viruses. Namely, dysregulated IFN-I expression can bring about severe pathology and disease. Multiple negative regulators have been verified affecting the expression and/or function of receptors, adaptors, enzymes, transcription factors, and effector proteins within IFN-I signaling. The role of VANGL2 as a core PCP component has been largely studied. However, little is known about its feature in antiviral innate immunity. We found that VSV infection can promote the expression of VANGL2, which is mainly dependent on IFN-I as IFN-I receptor (IFNAR)-KO mouse PEMs produced less VANGL2 than WT ones during VSV infection. Meanwhile, VANGL2 displays several functional features as the negative feedback to IFN-I production: (i) VANGL2 bounds the TBK1 ULD domain via its PkBD that is cytoplasmic facing; (ii) VANGL2 promotes K48-linked ubiquitination of TBK1 at K372 by recruiting TRIP to catalyze the ubiquitin chains of TBK1, thus enhancing OPTN-mediated autophagic degradation of TBK1 (Fig. 7G). In addition, we found that VANGL2-TBK1 is located in the cytosol, although VANGL2 has four consecutive transmembrane domains, suggesting that the cytoplasmic location of VANGL2 is similar to some other well-studied transmembrane proteins, including Tspan8, PD-L1, IFITM3, and ACE2 (*36*–*39*), and the underlying mechanism needs further investigation. Further in vivo studies found that mice with myeloid specific deletion of VANGL2 was beneficial to inhibit the replication of VSV due to stronger IFN-I responses, and these CKO mice were more resistant to lethal VSV infection than WT mice. In addition, we also substantiate the negative regulation of VANGL2 to IFN-I using different cell types in vitro, which was also confirmed through RNA sequencing (RNA-seq). Given this evidence, we define a previously undiscovered role of VANGL2 as a negative regulator in antiviral innate immunity.

TBK1 is a key kinase of both cGAS-STING and RLR-MAVS IFN-I signaling triggered by DNA and RNA viruses. Positive regulators like neuregulin receptor degradation protein 1 (Nrdp1), RING finger protein 128 (RNF128), tyrosine kinase Src, Raf kinase inhibitory protein (RKIP), Glycogen Synthase Kinase-3 Beta (GSK3β), DNA methyltransferases 3A (Dnmt3a), histone deacetylase 3 (HDAC3), and tripartite motif protein 9 (TRIM9) could enhance the autophosphorylation or activation, and USP1-UAF1 complex could stabilizes TBK1 to enhance its function. In contrast, regulators including A20/TAX1BP1, ubiquitin conjugating enzyme E2S (UBE2S), USP2b, cylindromatosis (CYLD), TCDD inducible poly[ADP-ribose] polymerase (TIPARP), Src family kinases Lck/Hck/Fgr, protein phosphatase 1B (PPM1B), protein phosphatase 4 (PP4), and cell division cycle-25a (Cdc25A) inactivate TBK1 and suppress IFN-I signaling (*40*). During viral infection, many negative regulators were reported to degrade TBK1, including E3 ligase, host factors, and viral factors. E3 ligase such as ankyrin repeat and SOCS box-containing 8 (ASB8), TRIP, DTX4, and NEDD4 is the basis for other host factors inducing ubiquitin-dependent degradation of TBK1. USP38 specifically cleaves K33-linked polyubiquitin chains from TBK1 at Lys^670^, allowing for subsequent K48-linked ubiquitination at the same position mediated by DTX4 and TRIP to degrade TBK1 (*8*). NLRP4 recruited the E3 ubiquitin ligase DTX4 to TBK1 for Lys^48^ (K48)–linked polyubiquitination at Lys^670^, which led to degradation of TBK1 (*41*). DYRK2 could phosphorylate Ser^527^ of TBK1, which is essential for recruiting NLRP4 and for the E3 ubiquitin ligase DTX4 to degrade TBK1 (*42*). Siglec1 associates with DNAX-activating protein of 12 kDa (DAP12) to recruit and activate the scaffolding function of src-homology 2-containing protein tyrosine phosphatase 2 (SHP2); SHP2 then recruits E3 ubiquitin ligase TRIM27, which induces TBK1 degradation via K48-linked ubiquitination at Lys^251^ and Lys^372^ (*33*). A recent study by Deng *et al.* (*9*) also found that TRAF3IP3 works as a negative regulator that interacts with endogenous TRAF3 and TBK1, which leads to the degradative K48 ubiquitination of TBK1 via its K372 residue in a DTX4-dependent fashion. There are many similarities between VANGL2 and the reported negative regulators. First, USP38, NLRP4, DYRK2, TRAF3IP3, and VANGL2 could directly interact with TBK1, which is different from Siglec1. Second, although these regulators promote the K48-linked ubiquitination of TBK1, the E3 ligases used are different. TRIP and DTX4 were identified to catalyze TBK1 K48-linked polyubiquitination at Lys^670^. K372 is vital for TRIP in our study and also necessary for DTX4. Besides, K372 is one of the two sites that are required for TRIM27. These findings indicated the target site in substrate that E3 ligases bind depends on the regulators. Third, consistent with the research about TRAF3IP3, we constructed the in vivo infection model using mice specifically lack VANGL2 in myeloid cells to verify the harmful role of VANGL2 in vivo, especially in macrophages, which differs from the study about USP38 that used USP38-KO mice. The last but important thing is that VANGL2-induced autophagic degradation is quite from others contributing to proteasome degradation. In general, VANGL2 is a regulator that not only has some parallels but also holds lots of distinctions from other reported regulators.

Notably, ubiquitination is an essential PTM for TBK1 to be activated or degraded. K63-linked polyubiquitination is critical for activating TBK1 (*5*), and K33-linked polyubiquitin chains on Lys^670^ of TBK1 may prevent the degradation of TBK1 (*8*). Besides the mentioned E3 ligase conjugating K48-linked polyubiquitination to TBK1 to induce its degradation, K27-linked polyubiquitination catalyzed by NEDD4 can also facilitate TBK1 autophagic degradation (*7*). We reported that VANGL2 could promote K48-linked polyubiquitination of TBK1 via TRIP to mediate autophagic degradation of TBK1, which is quite different from other published works that K48-linked polyubiquitination normally causes proteasomal degradation. However, increasing studies update this traditional opinion. K48 ubiquitination was first reported to be responsible for OPTN autophagic degradation by p62 in 2014 (*43*). Furthermore, Chen *et al.* (*44*) reported that K48-Ub–linked cGAS could be autophagic degraded by p62. In addition, Jin *et al.* (*37*) recently reported that ACE2 with K48-linked ubiquitination could be degraded by cargo receptor TOLLIP in a selective autophagy-dependent manner. In general, these works indicated that K48 ubiquitination is not only severing for proteasome and would bring us some insight into TBK1.

Proteasomal degradation of ubiquitin-tagging proteins has been recognized as a key determinant of protein stability. However, with the discovery of autophagic cargo receptors, which bind both ubiquitinated substrates and microtubule-associated protein 1A/1B-light chain 3 (LC3) modifier on the inner sheath of autophagosomes, selective autophagy is being indicated as a common way to degrade targeted protein. Regarding TBK1, earlier studies widely believed that TBK1 is degraded only via proteasomal degradation by negative regulators like NLRP4, DTX4, TRIP, TRIM27, USP38, NLRP14, and TRAF3IP3. However, a recent report showed that selective autophagy is also responsible for E3 ligase NEDD4 and SARS-CoV-2 NSP13 to degrade TBK1. Besides, the role of TBK1 in autophagy has been widely studied as TBK1 not only can phosphorylate syntaxin 17 to form the complex containing focal adhesion kinase family interacting protein of 200 kDa (FIP200), UNC51-like kinase 1 (ULK1), and autophagy-related gene 13 (ATG13) (*45*) but also can phosphorylate cargo receptors, including OPTN, p62, NDP52, and TAX1BP1, to promote selective autophagy. Given the intimate connection between TBK1 and autophagy, we believe that selective autophagy is essential in regulating the protein stability of TBK1. Actually, besides TBK1, proteins like ACE2 (*46*, *47*), p65 (*48*, *49*), cGAS (*44*, *50*), and MAVS (*51*, *52*) were also reported to be degraded by both ubiquitin-proteasome system and autophagy. Collectively, these studies suggested that protein degradation is complex and that the specific degradation of TBK1 depends on the context.

Selective autophagy degradation is distinguished from nonselective degradation as the mechanism behind selective autophagy must ensure valid recognition and sequestration of the cargo protein within autophagosomes (*53*). TBK1 has been reported to be recognized by cargo receptors like NDP52, TBC1 domain family member 9 (TBC1D9), and p62 during selective autophagy (*7*, *26*, *34*, *54*). However, whether OPTN recognizes TBK1 via K48-linked polyubiquitin was unclear. Published works have proved that OPTN can bind to ATG9A, LC3, MYO6, TBK1, and the ubiquitin chains of cargos to act on multiple steps during autophagy (*55*, *56*). Besides, OPTN can be phosphorylated by TBK1 on S177, S473, and S513 sites to enhance the binding of OPTN to multiple ubiquitin chains, which could promote the process of selective autophagy (*43*). Moreover, a glaucoma-associated mutated OPTN^E50K^ enhances its interaction with TBK1 but reduces autolysosomes (*57*), and polyubiquitin binding to OPTN is required for optimal activation of TBK1 and production of IFN-β (*58*). Given the inherent role of OPTN as a cargo receptor and its close interrelation with TBK1, these findings remind us that OPTN may be a potential cargo receptor for TBK1, which was verified by our study. Earlier studies showed that OPTN, containing a ubiquitin-binding domain present in A20-Binding Inhibitors of NF-κB (ABINs) and the ubiquitin binding domain (UBAN) in NF-κB essential modulator (NEMO) domain and zinc finger domain, prefers to bind to linear polyubiquitin chains and K63 chains, but not K48 chains, which were validated by pull-down assay and structurally analysis (*57*, *59*). However, HECT domain and ankyrin repeat containing E3 ubiquitin protein ligase 1 (HACE1) could conjugate K48 chains onto OPTN to induce OPTN autophagic degradation in a p62-dependent way, which proved the ability that OPTN interacts with K48 chains in a physiological condition (*43*). Here, we ascertain OPTN as the cargo receptor function on recognizing TBK1 with K48-linked polyubiquitin chains. Furthermore, we show that VANGL2 not only can combine with OPTN but also can enhance the interaction between OPTN and the substrate, TBK1, thus promoting the autophagic degradation of TBK1 during VSV infection.

SARS-CoV-2 has been identified as escaping from the human immune system via various mechanisms, including inhibition of PRR-mediated signaling cascades through viral protein or host-negative immune regulators (*60*). By analyzing the database, Large-scale single-cell analysis reveals critical immune characteristics of COVID-19 patients (http://covid19.cancer-pku.cn/) (*61*), we also discovered that about 7.36% of 258 patients with coronavirus disease 2019 (COVID-19) hold a higher expression of VANGL2 than healthy volunteers and the VANGL2 expression is highly correlated with the severity of COVID-19 in these patients. In addition, the expression of VANGL2 presented a reverse correlation with *IFITM3*, *ISG15*, *GBP1*, and *TRIM27*, which have been reported to play important roles in antiviral immunity in the COVID-19 patients’ database. All the above suggested VANGL2 may function in the progress of COVID-19, and the underlying mechanism needs to be further investigated.

We identified VANGL2 as an important negative regulator of antiviral IFN-I signaling by strengthening the selective autophagic degradation of TBK1. The core component of PCP, VANGL2, represents an “innate immune brake” for antiviral immunity, which can potentially to be an immune checkpoint. Our findings extend thoughts for regulating to innate immunity and reveal a previously uncharted character of VANGL2 in modulating antiviral innate immunity.

## MATERIALS AND METHODS

### Animal studies

All animal experiments were approved by the Southern Medical University Animal Care and Use Committee (SMUL20201010). All animal experiments were performed in specific pathogen–free levels using 6- to 8-week-old mice. Mice were kept with 50 to 60% humidity and daily cycles of 12 hours of light at an ambient temperature of 21° to 23°C. For mice breeding, *Vangl*2^fl/fl^ (stock no. 025174) and *Lyz2*-Cre (stock no. 4781) mice on a C57BL/6 background were obtained from the Jackson Laboratory (Las Vegas, NV, USA). *Vangl2*^fl/fl^ mice were crossed with *Lyz2*-Cre mice whose age and sex matched to obtain *Vangl2*^fl/fl^*Lyz2*-Cre^+^ mice with *Vangl2*-specific deficiency in myeloid cells. Cohoused littermate controls with normal *Vangl2* expression were used as controls.

### Cell isolation and culture

#### Cell line culture

Human embryonic kidney (HEK) 293T, A549, THP-1, and Vero cells were obtained from the American Type Culture Collection. Cell lines mentioned above were maintained in complete Dulbecco’s modified Eagle’s medium (DMEM) (Corning) supplemented with 10% fetal bovine serum (FBS) (HyClone), penicillin (100 U/ml), and streptomycin (100 μg/ml) (Sigma-Aldrich).

#### Isolation of mouse CD11b^+^ splenocytes

Spleens from WT or *Vangl2*^fl/fl^ *Lyz*2^+^ mice were subtly dissected and then digested in 2% FBS-DMEM containing collagenase type II (1 mg/ml; Sigma-Aldrich) and deoxyribonuclease I (200 U/ml; Sigma-Aldrich) at 37°C for 30 min. Tissues digested were filtered through a 70-μm cell strainer. After Ammonium Chloride-Potassium (ACK) blood lysis buffer removed red cells, the rest of splenocytes were labeled with anti-mouse CD11b biotin antibodies (BioLegend). The above mixture was incubated with streptavidin-paramagnetic particles (BD Biosciences) at 4°C for 30 min. Purification of CD11b^+^ splenocytes was performed by DynaMag (Thermo Fisher Scientific) and washed three times with 2% FBS–phosphate-buffered saline (PBS) buffer. Isolated cells achieved a purity of ≥95% and measured by fluorescence-activated cell sorting.

#### Isolation of mouse PEMs and NEs

Mouse PEMs were acquired from ascites of indicated mice, which were injected intraperitoneally with 4% Brewer thioglycollate medium (BD Biosciences) for three consecutive days before sacrifice. As for NEs, indicated mice were injected intraperitoneally with 4% Brewer thioglycollate medium (BD Biosciences) for 4 hours before sacrifice. Cold PBS was injected into the peritoneum of sacrificed mice. Then, fluid containing PEMs or NEs was aspirated from the peritoneum after the shake and collected after centrifugation at 800 rpm for 5 min.

#### Isolation of mouse BMDMs

Mouse bone marrow cells were isolated from the tibia and femur and cultured in L-929 conditional medium [70% DMEM, 10% FBS, 20% L-929 medium, penicillin (100 U/ml), and streptomycin (100 μg/ml)] for 5 days. All cells were grown in a 37°C incubator with 5% CO_2_.

### Plasmid construction and transfection

Plasmids for VANGL2 and TBK1 and its mutants were cloned into the pcDNA3.1 vector with an indicated tag for transient expression. IFN-β and ISRE promoter luciferase reporter plasmids and plasmids for cargo protein as well as TBK1-related K-R mutants were provided by J. Cui (Sun Yat-sen University), and mammalian expression plasmids for RIG-I, MAVS, cGAS, STING, TBK1, and IRF3 had been stored in our laboratory. Human-TRAF3IP3 CDS sequence was cloned into pcDNA3.1 FLAG-tag empty vector to generate FLAG-TRAF3IP3. HEK293T, A549, THP-1, and PBMC transfection was performed using Lipofectamine 2000 (Invitrogen) according to the protocols recommended by the manufacturer.

### Luciferase and reporter assays

HEK293T cells (1 × 10^5^) were plated in 24-well plates and transfected with plasmids encoding the IFN-β/ISRE luciferase reporter (Firefly luciferase) and pRL-TK (Renilla luciferase), together with different plasmids as follows: FLAG-cGAS, FLAG-STING, FLAG-RIG-I, FLAG-MAVS, FLAG-TBK1, FLAG-IRF3-5D, and FLAG-IKKi and an increasing dose of the HA-VANGL2 (0, 100, and 200 ng) or empty vector. The same number (1 × 10^5^) of transfected cells as indicated was then treated with or without VSV [multiplicity of infection (MOI) of 0.5], poly(I:C) (2 μg/ml) (InvivoGen, no. tlrlpic), poly(dA:dT) (2 μg/ml) (InvivoGen, no. ttlrl-patn), gDNA (2 μg/ml), or RNA (2 μg/ml) for 6 hours. Samples were collected at 24 to 36 hours after transfection, and luciferase activity was measured using the Dual-Luciferase Reporter Assay Kit (Promega) performed with a Luminoskan Ascent luminometer (Thermo Fisher Scientific). The activity of firefly luciferase was normalized by that of Renilla luciferase to obtain relative luciferase activity.

### Cell treatment

BMDMs, NEs, and A549 cells were treated with or without VSV (MOI of 0.5) for the indicated time to test cytokines, ISGs expression, and signaling pathway activation. For dual luciferase assay, cells were treated as described above. CHX (100 μg/ml) (Selleck) was used to block protein synthesis, as indicated in the figures. Rapamycin (250 nM) (Selleck) or EBSS (Thermo Fisher Scientific) was used for autophagy inducement, as indicated in the figures. For protein degradation inhibition assays in HEK293T cells, 3-MA (10 mM) (Selleck) or Baf A1 (0.2 μM) (Selleck) was used to inhibit autolysosome- or lysosome-mediated protein degradation, respectively. MG132 (10 μM) (Selleck) inhibited proteasome-mediated protein degradation. Z-VAD (50 μM) (Selleck) inhibited caspase-mediated protein degradation.

### Viral infection

VSV and VSV–enhanced green fluorescent protein replicate in Vero cells. Viral titers were determined by 50% of the tissue culture’s infectious dose. Cells were infected with viruses at the indicated time (MOI of 0.5). For in vivo viral infection, 6- to 8-week-old sex-matched mice were intravenously injected with the indicated dose of VSV. The weight and survival were monitored continuously. Cytokine secretion and tissue viral titers were measured at indicated postinfection time.

### Enzyme-linked immunosorbent assay

IFN-β in cell supernatants and mice serum was measured using an enzyme-linked immunosorbent assay kit (DY8234-05, R&D Systems) following the manufacturer’s instructions. Absorbance was detected at 450 nm by the Multiskan FC (Thermo Fisher Scientific).

### IP and IB analysis

Cells were lysed by low-salt lysis buffer. For endogenous IP, whole-cell lysates were treated with indicated antibodies overnight and then incubated with protein A and G beads (Pierce) for 4 to 6 hours. For exogenous IP, whole-cell lysates were only incubated with anti-FLAG or anti-MYC (EQKLISEEDL) agarose gels. Immunoprecipitates were eluted with 2× SDS loading buffer after washing five times with low-salt lysis buffer. Cells were lysed by low-salt lysis buffer, boiled for 5 min with SDS loading buffer (Cell Signaling Technology), and resolved on SDS–polyacrylamide gel electrophoresis gels. Proteins were transferred to a polyvinylidene difluoride membrane (MilliporeSigma). The membranes were blocked with 5% (w/v) reagent-grade nonfat milk and further incubated with the antibodies as listed in the “Antibodies” section. EMD Millipore Luminata Western HRP Chemiluminescence Substrate was used for protein detection for all blots.

### IF and confocal microscopy

Mouse primary BMDMs or PEMs cultured on glass coverslips were treated with VSV or not as indicated in the figures. The cells were washed three times using PBS, fixed with 4% paraformaldehyde (diluted in PBS) for 20 min, permeabilized with 0.2% Triton X-100 for 20 min, and then blocked with 3% BSA for 30 min at room temperature. The cells were then stained with the corresponding antibodies for 8 to 12 hours at 4°C, followed by incubation with fluorescent dye–conjugated secondary antibodies for 1 hour at room temperature. The nuclei were counterstained with 4′,6-diamidino-2-phenylindole (Sigma-Aldrich) for 5 min before being subjected to confocal microscopy observation.

### Antibodies

Primary antibodies used for IP and immunoblot (IB) analysis are as follows: anti-TRAF3IP3 (no. ab243711, Abcam), anti-VANGL2 (C-2) (no. sc-515187, Santa Cruz Biotechnology), anti–phosphor-TBK1/NAK (Ser^172^) (no. 5483, Cell Signaling Technology), anti-TBK1/NAK (no. 3013, Cell Signaling Technology), anti–phosphor-IRF3 (Ser^396^) (no. 4947, Cell Signaling Technology), anti-IRF3 (no. 11904, Cell Signaling Technology), anti-FLAG (M2) (no. A8592, Sigma-Aldrich), anti-hemagglutinin (HA) (C29F4) (no. 5017, Cell Signaling Technology), anti-MYC (9B11) (no. 2276, Cell Signaling Technology), anti-p62 (D5L7G) (no. 88588, Cell Signaling Technology), anti-OPTN (EPR20654) (no. ab213556), anti-NDP52 (no. 12229-1-AP, Proteintech), anti-TOLLIP (no. 11315-1-AP, Proteintech), anti-ATG5 (no. 10181-2-AP, Proteintech), anti-BECLIN1 (no. 11306-1-AP, Proteintech), anti–Na,K-depenent adenosine triphosphatase (Na,K-ATPase) (D4Y7E) (no. 23565, Cell Signaling Technology), anti-Ub (P4D1) (no. 3936, Cell Signaling Technology), anti-K48-Ub (D9D5) (no. 8081, Cell Signaling Technology), anti-TUBULIN (210-444h) (no. sc-5274, Santa Cruz Biotechnology), and anti–glyceraldehyde-3-phosphate dehydrogenase (G-9) (no. sc-365062, Santa Cruz Biotechnology). Primary antibodies used for immunofluorescence (IF) analysis are as follows: TBK1 (A-6) (no. sc-398366, Santa Cruz Biotechnology), VANGL2 (2G4) (no. MABN750, Merck Life Sciences), and LAMP1 (D2D11) (no. 9091, Cell Signaling Technology). Secondary antibodies used for IB analysis are as follows: goat anti-rabbit immunoglobulin G (IgG) H&L horseradish peroxidase (no. ab6721, Abcam), and goat anti-mouse IgG H&L GRP (no. ab6789, Abcam). Secondary antibodies used for IF analysis are as follows: goat anti-mouse IgG H&L AF594 (no. ab150116, Abcam), goat anti-rat IgG H&L AF488 (no. ab150165, Abcam), and goat anti-rabbit IgG H&L AF647 (no. ab150079, Abcam). Flow sorting antibodies are as follows: CD3 (17A2) (no. 11-0032-82, eBioscience), CD19 (6D5) (no. 115507, BioLegend), and CD11b (M1/70) (no. 48-0112-82, eBioscience).

### Cellular fractionation

The Cell Fractionation Kit (no. 9038, Cell Signaling Technology) was used for cell fractionation according to the manufacturer’s protocol and the published works (*23*, *24*). Briefly, cells were collected by scraping, washed in PBS, and pelleted (350*g*, 5 min). The counted cells were resuspended in 500 μl of PBS and 100 μl was reserved for whole-cell lysis in low-salt lysis buffer, and the remaining cell pellet was subjected to cell fractionation. Cytoplasmic proteins were isolated using Cytoplasm Isolation Buffer (CIB) buffer, and integral membrane and organellular membrane proteins were isolated using Membrane Isolation Buffer (MIB). Cytosol-located TUBULIN and cytomembrane-located Na^+^K^+^-ATPase were used to demonstrate the efficiency of the cellular fractionation.

### RNA preparation and qPCR

Total RNA was purified from splenic tissue, lymph node, or stimulated cells through the TRIzol reagent (Invitrogen). Moreover, the complementary DNA (cDNA) was generated using a Starscript II first-stand cDNA synthesis kit (GenStar). Real-time PCR was performed on QuantStudio 6 flex (Thermo Fisher Science) using a RealStar green power mixture (GenStar) with primers as listed in table S1.

### Library preparation and RNA-seq

Total RNA extracted from BMDMs with indicated treatment was subjected to RNA-seq. The RNA quality was determined by 2100 Bioanalyzer (Agilent) and quantified using the ND-2000 (NanoDrop Technologies). RNA-seq transcriptome library was prepared following the TruSeqTM RNA sample preparation Kit from Illumina (San Diego, CA, USA) using 1 μg of total RNA, and the paired-end RNA-seq sequencing library was sequenced with the Illumina HiSeq xten/NovaSeq 6000 sequencer (2 × 150–base pair read length).

### DEG analysis and functional enrichment

The raw paired-end reads were trimmed and quality controlled by SeqPrep (https://github.com/jstjohn/SeqPrep) and Sickle (https://github.com/najoshi/sickle) with default parameters (*62*). To identify DEGs (differentially expressed genes) between two different samples, the expression level of each transcript was calculated according to the transcripts per million reads method. RSEM (http://deweylab.biostat.wisc.edu/rsem/) (*63*) was used to quantify gene abundances. The DEGs were identified with a *P* value of ≤0.05 and fold change of ≥2 as the significance threshold. In addition, GO functional enrichment analysis was performed to identify which DEGs were significantly enriched in GO terms and metabolic pathways at Bonferroni-corrected *P* value of ≤0.05 compared with the whole-transcriptome background. GO functional enrichment was carried out by Goatools (https://github.com/tanghaibao/Goatools) and KOBAS (http://kobas.cbi.pku.edu.cn/home.do) (*64*).

### Statistical analyses

All statistical analyses were performed using GraphPad Prism version 9.0 (GraphPad software). The data of all quantitative experiments are presented as means ± SD of at least three independent experiments. All statistical analyses were carried out with Student’s *t* test. Data are represented as means ± SD.

## Notes

View/request a protocol for this paper from *Bio-protocol*.
