# Supplementary material for: VANGL2 inhibits antiviral IFN-I signaling by targeting TBK1 for autophagic degradation
Source: Sci Adv. 2023 Jun 23;9(25):eadg2339. doi: 10.1126/sciadv.adg2339 (PMC10289648; doi:10.1126/sciadv.adg2339)
Supplement: Supplementary file 1 — Figs. S1 to S7 Table S1 [file sciadv.adg2339_sm.pdf]

Science Advances
AAAS

Supplementary Materials for

VANGL2 inhibits antiviral IFN-I signaling by targeting TBK1 for autophagic degradation

Zhiqiang Hu *et al.*

Corresponding author: Xiao Yu, xiaoyu523@smu.edu.cn

Sci. Adv. **9**, eadg2339 (2023)
DOI: 10.1126/sciadv.adg2339

This PDF file includes:

Figs. S1 to S7
Table S1

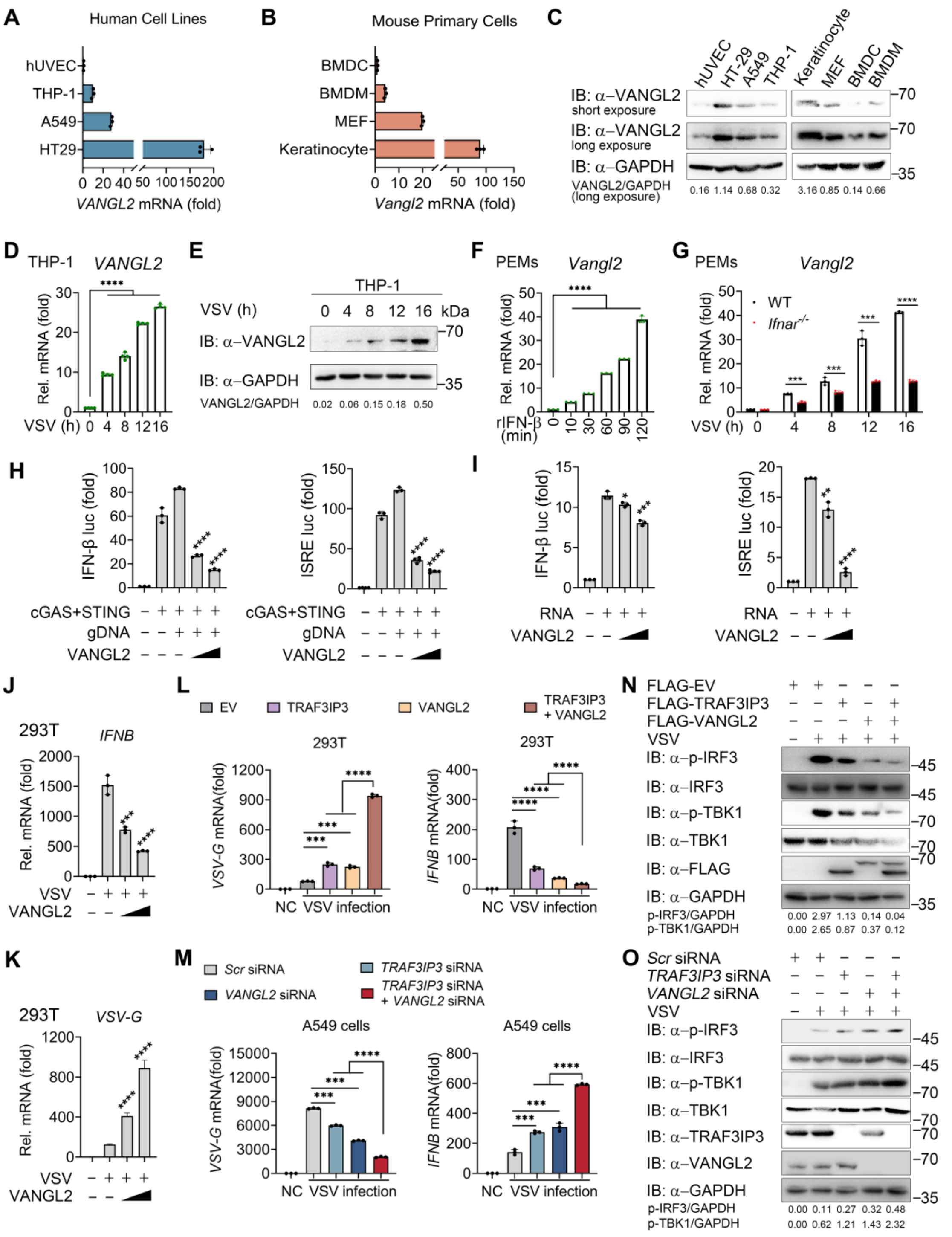

Fig. S1. Virus- and IFN-I- induced VANGL2 negatively regulates antiviral immunity. (**A** to **C**) VANGL2 expression between indicated human cell lines (A and C)

and primary mouse cells (B and C) (**D** and **E**) qRT-PCR (D）and immunoblotting (E) analysis of VANGL2 mRNA and protein level changed in THP-1 cells infected with VSV (MOI=0.5) for 0-16 h. (**F**) qRT-PCR analysis of VANGL2 mRNA level changed in mouse primary PEMs stimulated with recombinant IFN-β (10 ng/ml) at indicated time points. (**G**) qRT-PCR analysis of VANGL2 mRNA level in WT and *Ifnar*$^{-/-}$ PEMs infected with VSV (MOI=0.5) for the indicated times. (**H** and **I**) Luciferase reporter assays analyzing IFN-β or ISRE promoter activity of HEK293T cells transfected with increasing amounts (wedge represents 300 and 500 ng) of HA-VANGL2 or empty vector (EV) for 24 h, followed by treatment with or without exogenous gDNA (H), or RNA (I) for 12 h respectively. (**J** and **K**) qRT-PCR analysis of *IFNB* (J) and *VSV-G* (K) mRNA expression in HEK293T cells transfected with HA-VANGL2 for 24 h, then infected with VSV (MOI=0.5) for 18 h. (**L** and **M**) qPCR test for *VSV-G* as well as *IFNB* mRNA levels in TRAF3IP3, VANGL2 or both overexpressed 293T cells (L), and TRAF3IP3, VANGL2 or both silenced A549 cells (M) after VSV infection for 8 h. (**N** and **O**) Immunoblotting analysis of indicated protein levels in TRAF3IP3, VANGL2 or both overexpressed 293T cells (N), and TRAF3IP3, VANGL2 or both silenced A549 cells (O) after VSV infection for 8 h. Data with error bars are represented as mean ± SD. Each panel is a representative experiment of at least three independent biological replicates. *$P<0.05$, **$P<0.01$, ***$P<0.001$ and ****$P<0.0001$ as determined by unpaired Student's *t*-test.

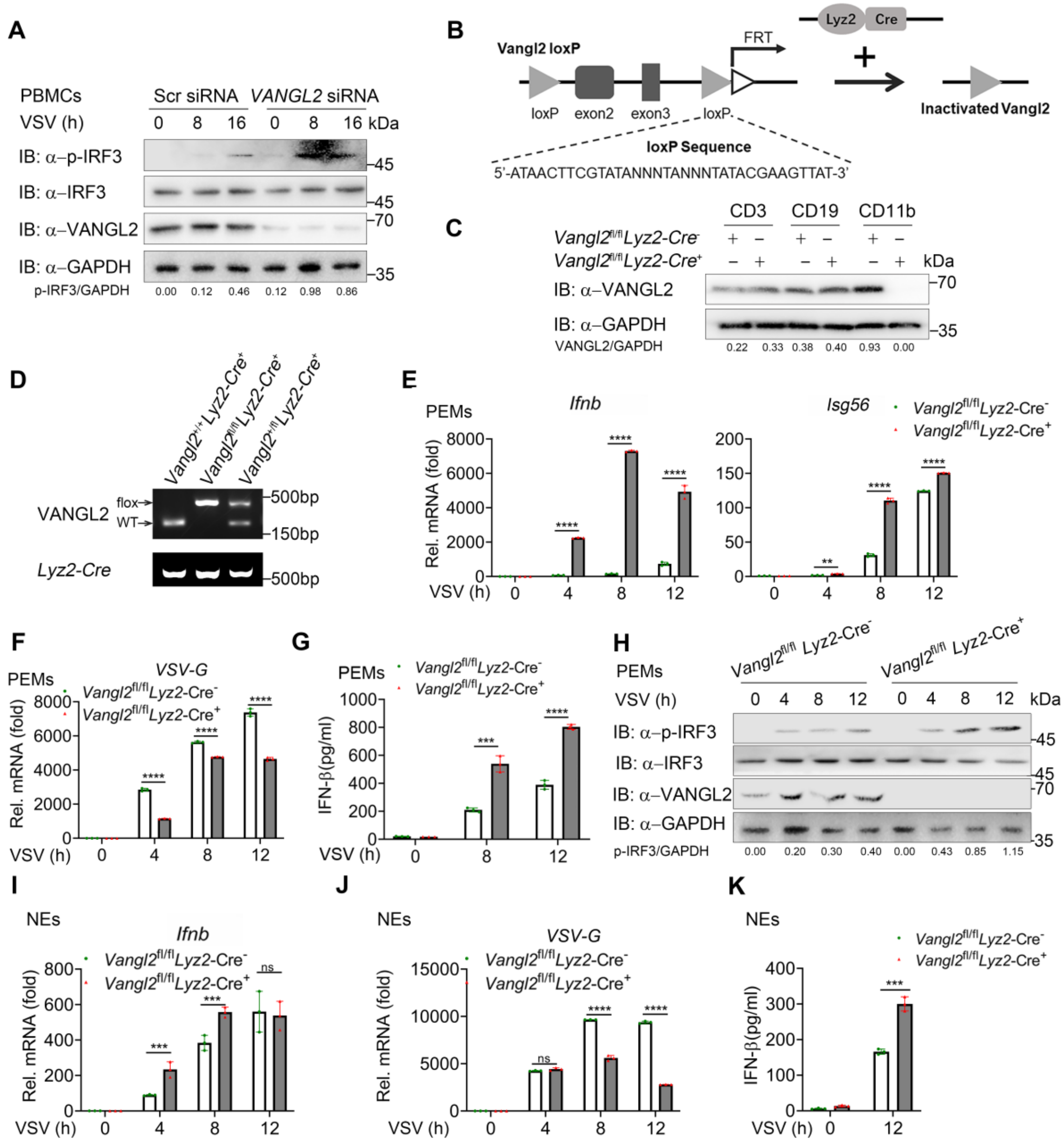

Fig. S2. VANGL2 ablation facilitates antiviral response in primary mouse cells. (**A**) Immunoblotting analysis of total and phosphorylated IRF3 using PBMCs transfected with *Scr* siRNA or *VANGL2* specific siRNA, followed by VSV (MOI=0.5) infection at indicated time points. (**B**) Control (*Vangl2*$^{fl/fl}$ *Lyz2*-Cre^{-}) or mice with *Vangl2* ablation in myeloid cells (*Vangl2*$^{fl/fl}$ *Lyz2*-Cre^{+}, CKO) were generated as shown. (**C** and **D**) Immunoblotting (C) of flow-sorted CD3^{+} T cells, CD19^{+} B cells, and

CD11b$^+$ myeloid cells and genotyping analysis (D) from indicated mice. (**E** to **K**) RT-PCR analysis, IFN-β ELISA, and immunoblotting analysis of indicated indexes using *Vangl2*$^{fl/fl}$ *Lyz2*-Cre$^-$ and *Vangl2*$^{fl/fl}$*Lyz2*-Cre$^+$ PEMs (E to H) or Neutrophils (I to K) infected with VSV (MOI=0.5) for the indicated times. Data with error bars are represented as mean ± SD. Each panel is a representative experiment of at least three independent biological replicates. ***P*<0.01, ****P*<0.001 and *****P*<0.0001 as determined by unpaired Student's *t*-test. ns, not significant

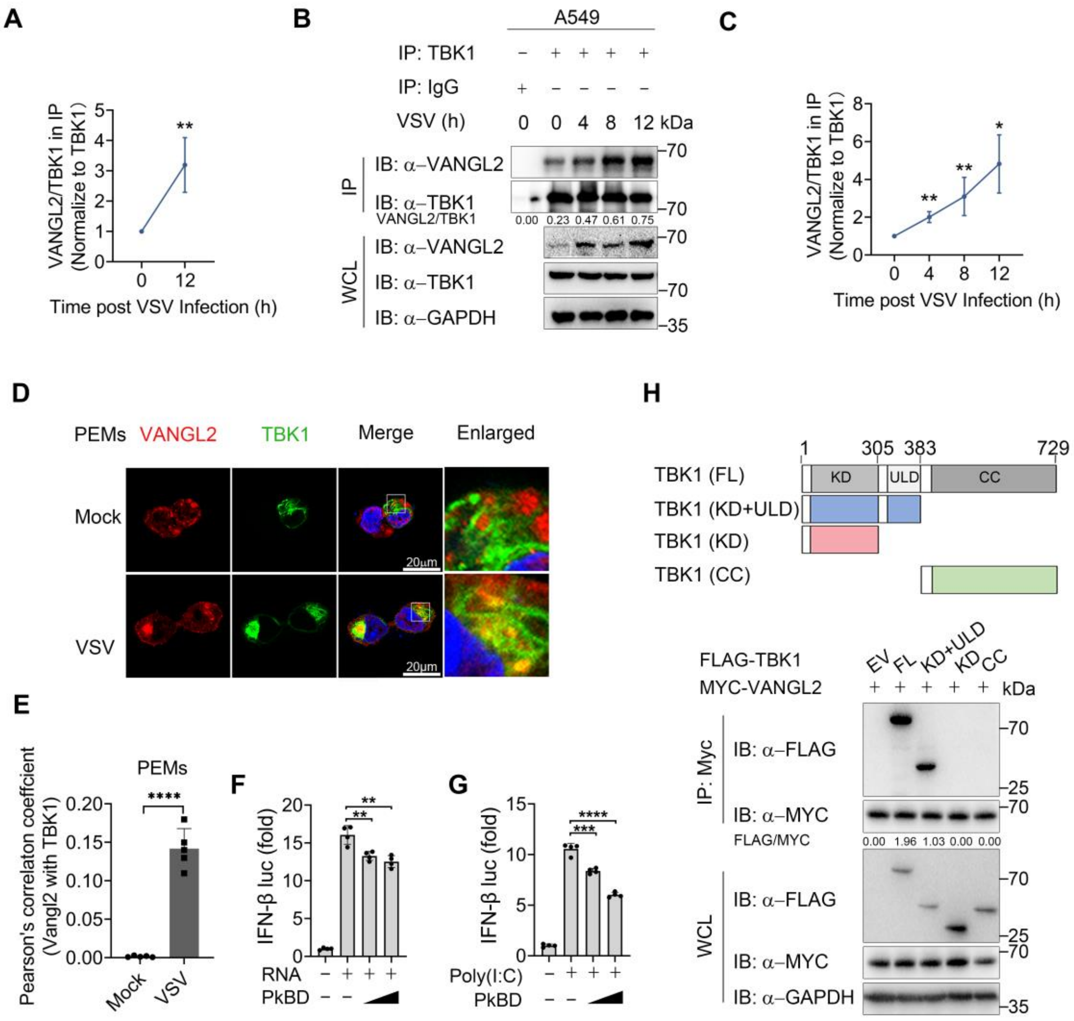

Fig. S3. VANGL2 PkBD domain binds to the ULD of TBK1. (**A**) Quantification of the VANGL2/TBK1 in immunoprecipitation shown in Fig. 3D. (**B**) Immunoblotting of endogenous proteins with the indicated antibodies using protein lysates of A549 cells infected with VSV (MOI=0.5) for the indicated times was subjected to immunoprecipitation with anti-TBK1 antibody. (**C**) Quantification of the VANGL2/TBK1 in immunoprecipitation shown in (B). (**D**) Mouse PEMs were infected with VSV (MOI=0.5) for 12 h before being harvested, then labeled with the indicated specific antibodies and analyzed via confocal microscopy. Red: VANGL2

signal; Green: TBK1 signal; Blue: DAPI (nuclei signal). Scale bars, 20 μm. **(E)** Quantitative analysis of the colocalization in (D). (**F** and **G**) Luciferase reporter assays analyzing IFN-β promoter activity of HEK293T cells transfected with increasing amounts (wedge represents 300 and 500 ng) of VANGL2 deletion construct HA tagged PkBD or empty vector (EV) followed by treatment with exogenous RNA (F) or poly(I:C) (G) for 12 h respectively. (**H**) Coimmunoprecipitation (IP, with anti-FLAG) and immunoblotting analysis using lysates from HEK293T cells transfected with FLAG-TBK1 and its deletions along with MYC-VANGL2. Data with error bars are represented as mean ± SD. Each panel is a representative experiment of at least three independent biological replicates. **P<0.01, ***P<0.001 and ****P<0.0001 as determined by unpaired Student's t-test.

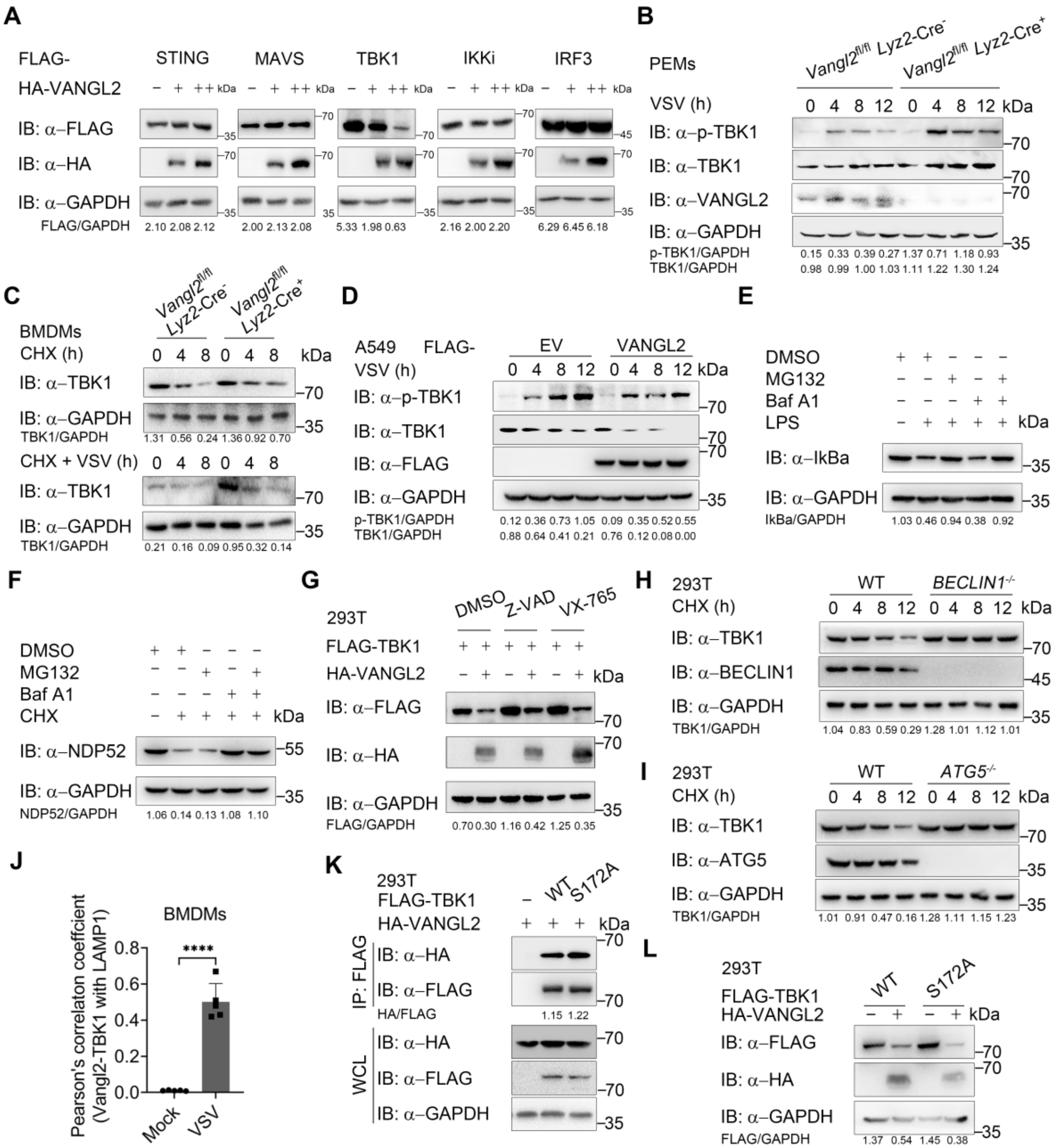

Fig. S4. VANGL2 specially degradates unphosphorylated TBK1 via autophagy. (**A**) Immunoblotting analysis using lysates from HEK293T cells transfected with the indicated FLAG-tagged expression vectors and HA-VANGL2. (**B**) Immunoblotting analysis of total and phosphorylated TBK1 using lysates from *Vangl2*$^{fl/fl}$ *Lyz2*-Cre^{-} and *Vangl2*$^{fl/fl}$ *Lyz2*-Cre^{+} PEMs, followed by infected with VSV (MOI=0.5) for the

indicated times. (**C**) Immunoblotting analysis of protein extracts of *Vangl2*$^{fl/fl}$ *Lyz2*-Cre^{-} and *Vangl2*$^{fl/fl}$ *Lyz2*-Cre^{+} BMDMs treated with CHX (100 μg/mL) at indicated time points, with or without VSV (MOI=0.5) infection. (**D**) Immunoblotting analysis of total and phosphorylated TBK1 using lysates from A549 cells transfected with FLAG-EV or FLAG-VANGL2 for 24 h, followed by infected with VSV (MOI=0.5) for the indicated times. **(E)** Immunoblotting analysis of protein extracts of THP-1 cells treated with LPS (50 ng/mL) for 2 h, followed by treatment with MG132 (10 μM), Baf A1 (0.2 μM) or both for 6 h. **(F)** Immunoblotting analysis of protein extracts of HEK293T cells treated with CHX (100 μg/mL) for 12 h, followed by treatment with MG132 (10 μM), Baf A1 (0.2 μM) or both for 6 h. (**G**) Immunoblotting analysis using cell lysates from HEK293T transfected with FLAG-TBK1, and EV or HA-VANGL2 for 24 h, followed by treated with DMSO, Z-VAD, VX-765 for 6 h. (**H** and **I**) Immunoblotting analysis of protein extracts of WT, BECLIN1 KO (H) or ATG5 KO (I) HEK293T cells treated with CHX (100 μg/mL) at indicated time points. **(J)** Quantitative analysis of the colocalization in Fig. 4K. (**K**) Coimmunoprecipitation (IP, with anti-FLAG) and immunoblotting analysis using lysates from HEK293T cells transfected with WT FLAG-TBK1 or FLAG-TBK1 S172A mutant, together with HA-VANGL2. (**L**) Immunoblotting analysis of protein lysates of 293 T cells transfected with vectors expressing WT FLAG-TBK1 or FLAG-TBK1 S172A mutant, together with HA-EV or HA-VANGL2. Each panel is a representative experiment of at least three independent biological replicates.

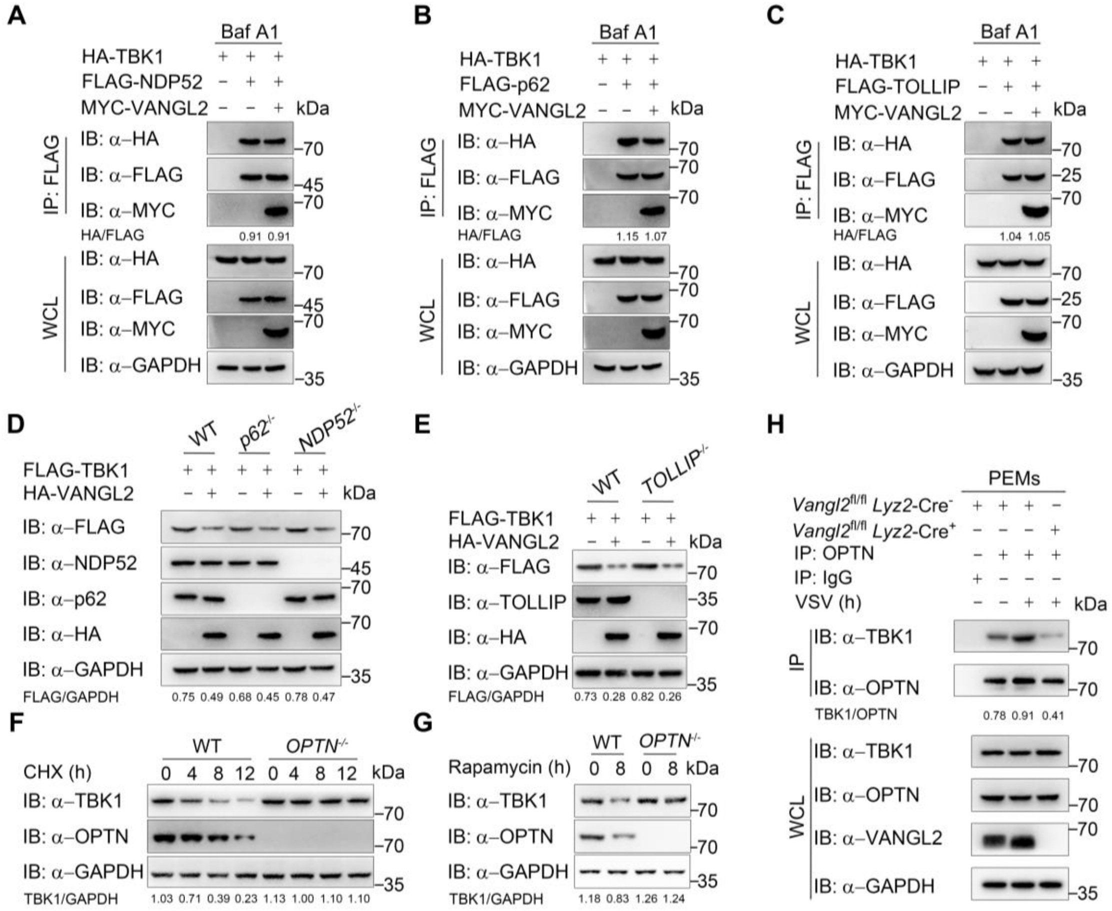

Fig. S5. Autophagic degradation of TBK1 induced by VANGL2 dependents on the cargo receptor OPTN, but not p62, NDP52 and TOLLIP. (**A** to **C**) Cell lysates were harvested after Baf A1 (0.2 μM) treatment (6 h) for coimmunoprecipitation (IP, with anti-FLAG) and immunoblotting analysis of HEK293T cells transfected with indicated FLAG-tagged cargo receptor NDP52 (A), p62 (B) or TOLLIP (C), and HA-TBK1, together with MYC-VANGL2. (**D** and **E**) Immunoblotting analysis of WT, *p62* KO (D), *NDP52* KO (D), or *TOLLIP* KO (E) HEK293T cells transfected with FLAG-TBK1, together with HA-EV or HA-VANGL2 for 24 h. (**F** and **G**) Immunoblotting analysis of protein extracts of WT, *OPTN* KO HEK293T cells treated with CHX (100 μg/mL) (F) or rapamycin (250 nM)

(G) at indicated time points. (**H**) *Vangl2*$^{fl/fl}$ *Lyz2*-Cre^{-} and *Vangl2*$^{fl/fl}$ *Lyz2*-Cre^{+} PEMs cells were infected with VSV (MOI=0.5), protein lysates were harvested for immunoprecipitation using an anti-OPTN antibody. Each panel is a representative experiment of at least three independent biological replicates.

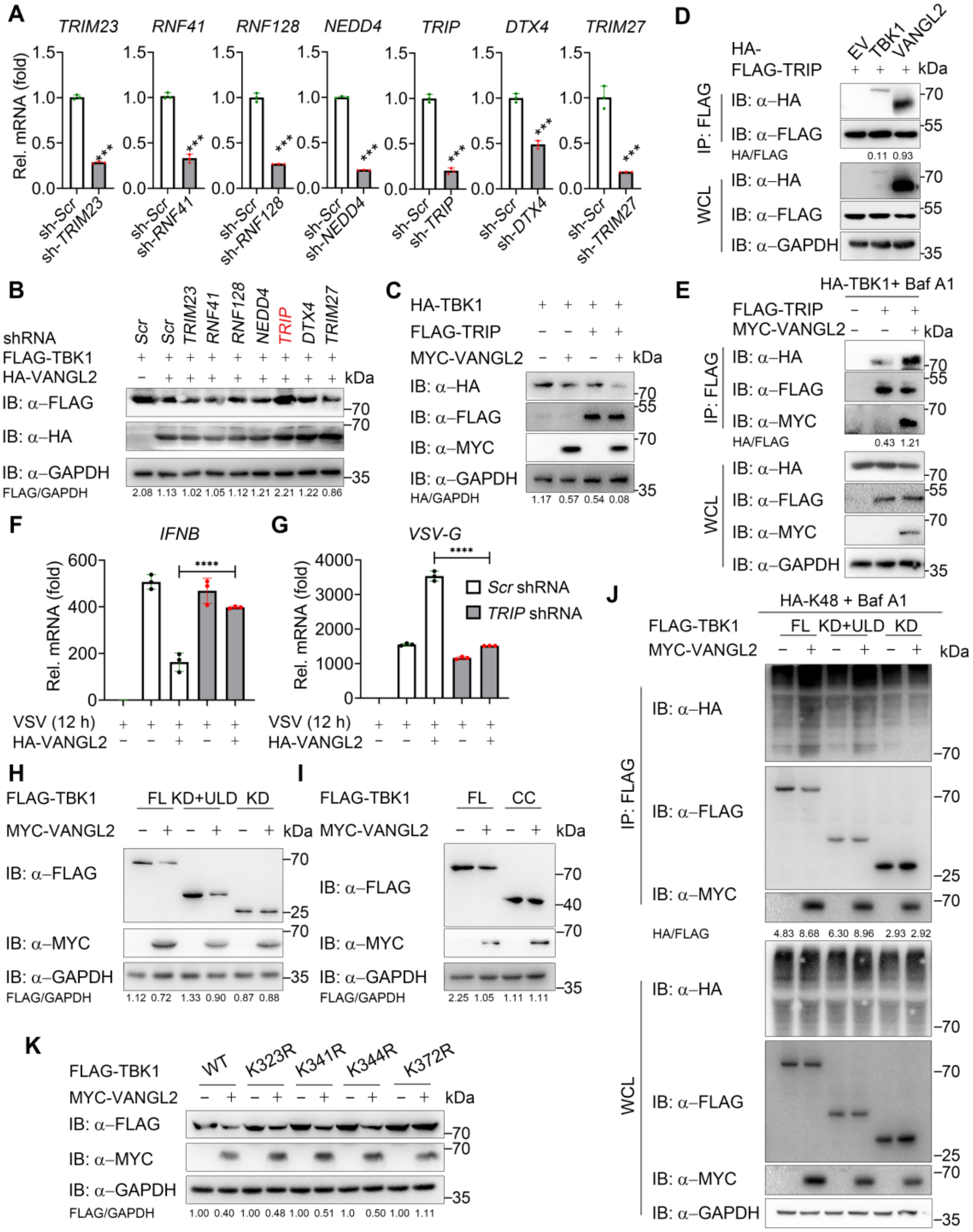

Fig. S6. TRIP-catalyzed K48-linked poly-ubiquitination at Lys372 of TBK1 is critical for VANGL2-mediated TBK1 degradation. (**A**) RT-PCR analysis of HEK293T cells transfected with *Scr* shRNA or E3 ligase-specific shRNAs for 24 h.

(**B**) Immunoblotting analysis of HEK293T cells transfected with *Scr* shRNA or E3 ligase specific shRNAs for 24 h, then transfected with FLAG-TBK1 and HA-EV or HA-VANGL2 for 24 h. (**C**) Immunoblotting analysis using lysates from HEK293T cells transfected with the indicated expression vectors. (**D**) Coimmunoprecipitation (IP, with anti-FLAG) and immunoblotting analysis using lysates from HEK293T cells transfected with FLAG-TRIP along with HA-tagged TBK1 or VANGL2. (**E**) Cell lysates were harvested after Baf A1 (0.2 μM) treatment (6 h) for coimmunoprecipitation (IP, with anti-FLAG) and immunoblotting analysis of HEK293T cells transfected with indicated vectors. (**F** and **G**) RT-PCR analysis of IFNB (F) or VSV-G (G) mRNA level changed in HEK293T cells transfected with Scr shRNA or TRIP specific shRNA for 24 h, followed by transfected with HA-EV or HA-VANGL2 for 24 h, before harvesting, the cells were infected with VSV (MOI=0.5) for 12 h. (**H** and **I**) Immunoblotting analysis of HEK293T cells transfected with full-length (FL) or deletion mutants of FLAG-TBK1, together with MYC-EV or MYC-VANGL2. (**J**) Coimmunoprecipitation (IP, with anti-FLAG) and immunoblotting analysis of HEK293T cells transfected with FL or deletions of FLAG-TBK1, together with HA-K48-linked ubiquitin and MYC-EV or MYC-VANGL2, protein lysates were harvested after Baf A1 (0.2 μM) treatment for 6 h. (**K**) Immunoblotting analysis of HEK293T cells transfected with MYC-VANGL2, with WT FLAG-TBK1 or its mutants. Each panel is a representative experiment of at least three independent biological replicates. ***P<0.001 and ****P<0.0001 as determined by unpaired Student's t-test.

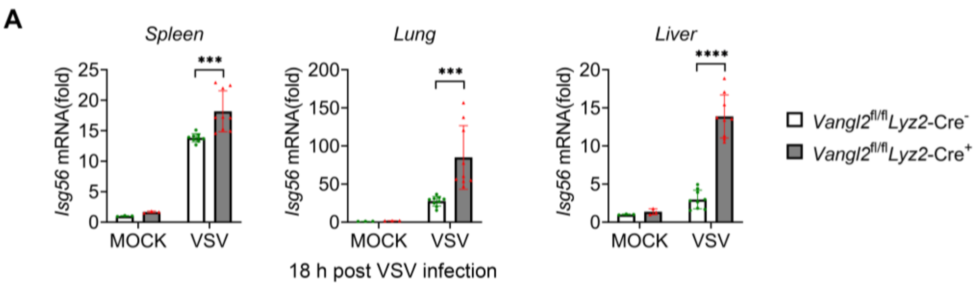

Fig. S7. VANGL2 deficiency promotes the expression of the antiviral gene. (**A**) RT-PCR analysis of *Isg56* mRNA in the spleen (left), lungs (center), and liver (right) from *Vangl2*$^{fl/fl}$ *Lyz2*-Cre^{-} and *Vangl2*$^{fl/fl}$ *Lyz2*-Cre^{+} mice treated with PBS or infected with VSV (1×10^{8} PFU per mouse) via intraperitoneal (*i.p.*) injection for 18 h. Each group is a representative experiment of at least 5 mice. ***P<0.001 and ****P<0.0001 as determined by unpaired Student's *t*-test.

Table. S1. Primers used in this study.

**Primer sequences for qRT-PCR in human**	
**Name**	**Sequences (5’ → 3’)**
IFNB1-F	GCTTGGATTCCTACAAAGAAGCA
IFNB1-R	ATAGATGGTCAATGCGGCGTC
ISG15-F	CGCAGATCACCCAGAAGATCG
ISG15-R	TTCGTCGCATTTGTCCACCA
ISG56-F	TTGATGACGATGAAATGCCTGA
ISG56-R	CAGGTCACCAGACTCCTCAC
VANGL2-F	AATCCCGAAAAGAAGGCTGT
VANGL2-R	CCCTATTCCCCACAACACAC
DTX4-F	GCCACCTTGAATCGTAC
DTX4-R	GGTTGACAGGACTGGA
TRIM27-F	AGCCTGATCGCTCAGCTAGAAG
TRIM27-R	GGAGGTGTGATCCAAG
TRAIP-F	CGCTGGAAGAACGCAA
TRAIP-R	GTGCTTGTTTGGTCTCAT
RNF128-F	TGGTTTCATCCATTCAGCAA
RNF128-R	AGTTTGAAGCTTCATCCACCA
NEDD4-F	TCAGGACAACCTAACAGATGCT
NEDD4-R	TTCTGCAAGATGAGTTGGAACAT
TRIM23-F	TGGTTGTAAACAAGCTCGGAG
TRIM23-R	ACTCTAGCACCTTCACTACAGC
RNF41-F	AACGCTGTGTTCGGCTGTAG
RNF41-R	AGGTGCTTAATGCAGTTATGGTT
GAPDH-F	GGAGCGAGATCCCTCCAAAAT
GAPDH-R	GGCTGTTGTCATACTTCTCATGG
**Primer sequences for qRT-PCR in mouse**	
**Name**	**Sequences (5’ → 3’)**

Ifnb-F	TCACCTACAGGGCGGACTTC
Ifnb-R	GGTGTCCGTGACTAACTCCAT
Isg15-F	GGTGTCCGTGACTAACTCCAT
Isg15-R	CTGTACCACTAGCATCACTGTG
Isg56-F	TGCGATCCACAGTGAACAAC
Isg56-R	ACTTCCGGGAAATCGATGAG
Vangl2-F	TGAGGGCCTCTTCATCTCC
Vangl2-R	GCCCGTGGAGTTAATTGGT
Gapdh-F	AAGGTCATCCCAGAGCTGAA
Gapdh-R	CTGCTTCACCACCTTCTTGA
**qRT-PCR Primer sequences for virus detection**	
**Name**	**Sequences (5’ → 3’)**
VSV-G-F	CAAGTCAAAATGCCCAAGAGTCACA
VSV-G-R	TTTCCTTGCATTGTTCTACAGATGG
**shRNA target sequences:**	
TRIM27 shRNA	AGGGCTGAAAGAATCAGGATC
TRIP shRNA	CCAGCATGGTTACTACGAAAC
DTX4 shRNA	TAAGGCAGCCGTGGTCAATGC
TRIM23 shRNA	GCAGTCATAGAGACAGAATTA
RNF41 shRNA	CCTGGAGGAGACAATTGAATA
RNF128 shRNA	TCTTAACGTGCAACCATATTT
NEDD4 shRNA	TGCAAGCACAACGTGCATTTA
Scr shRNA	AACAAGATGAAGAGCACCAAC
**siRNA sequences:**	
VANGL2 siRNA	UCCCAAGUCACACAAGUUUTT
Scr siRNA	CGUUAAUCGCGUAUAAUACGCGUA
**FLAG-human-TRAF3IP3 construction:**	
Forward:	cttggtaccgagctcggatccATGATCAGCCCAGACCCCA
Reverse:	tgctggatatctgcagaattcTCAGATCATCAGGTTGTCTTTATTGG
